# Boosting BCG with proteins or rAd5 does not enhance protection against tuberculosis in rhesus macaques

**DOI:** 10.1038/s41541-019-0113-9

**Published:** 2019-05-28

**Authors:** Patricia A. Darrah, Robert M. DiFazio, Pauline Maiello, Hannah P. Gideon, Amy J. Myers, Mark A. Rodgers, Joshua A. Hackney, Thomas Lindenstrom, Thomas Evans, Charles A. Scanga, Victor Prikhodko, Peter Andersen, Philana Ling Lin, Dominick Laddy, Mario Roederer, Robert A. Seder, JoAnne L. Flynn

**Affiliations:** 1 Vaccine Research Center, National Institute of Allergy and Infectious Diseases (NIAID), National Institutes of Health (NIH), Bethesda, MD USA; 2 Department of Microbiology and Molecular Genetics, University of Pittsburgh School of Medicine, Pittsburgh, PA USA; 3 Center for Vaccine Research, Statens Serum Institut, Copenhagen, Denmark; 4 Aeras, Rockville, MD USA; 5 Department of Pediatrics, Children’s Hospital of the University of Pittsburgh of UPMC, Pittsburgh, PA USA

**Keywords:** Immunology, Microbiology, Diseases

## Abstract

Tuberculosis (TB) is the leading cause of death from infection worldwide. The only approved vaccine, BCG, has variable protective efficacy against pulmonary TB, the transmissible form of the disease. Therefore, improving this efficacy is an urgent priority. This study assessed whether heterologous prime-boost vaccine regimens in which BCG priming is boosted with either (i) protein and adjuvant (M72 plus AS01_E_ or H56 plus CAF01) delivered intramuscularly (IM), or (ii) replication-defective recombinant adenovirus serotype 5 (Ad5) expressing various *Mycobacterium tuberculosis* (Mtb) antigens (Ad5(TB): M72, ESAT-6/Ag85b, or ESAT-6/Rv1733/Rv2626/RpfD) administered simultaneously by IM and aerosol (AE) routes, could enhance blood- and lung-localized T-cell immunity and improve protection in a nonhuman primate (NHP) model of TB infection. Ad5(TB) vaccines administered by AE/IM routes following BCG priming elicited ~10–30% antigen-specific CD4 and CD8 T-cell multifunctional cytokine responses in bronchoalveolar lavage (BAL) but did not provide additional protection compared to BCG alone. Moreover, AE administration of an Ad5(empty) control vector after BCG priming appeared to diminish protection induced by BCG. Boosting BCG by IM immunization of M72/AS01_E_ or H56:CAF01 elicited ~0.1–0.3% antigen-specific CD4 cytokine responses in blood with only a transient increase of ~0.5–1% in BAL; these vaccine regimens also failed to enhance BCG-induced protection. Taken together, this study shows that boosting BCG with protein/adjuvant or Ad-based vaccines using these antigens, by IM or IM/AE routes, respectively, do not enhance protection against primary infection compared with BCG alone, in the highly susceptible rhesus macaque model of tuberculosis.

## Tuberculosis vaccination: Multi-platform/route BCG boosting in rhesus macaques

BCG is the only clinically-approved tuberculosis (TB) vaccine but has highly variable protection against pulmonary manifestations of the disease. JoAnne Flynn and colleagues at the University of Pittsburgh investigate whether heterologous boosting can improve the efficacy of BCG vaccination in a highly susceptible rhesus macaque model. The boosting is performed using a variety of vaccination routes (intramuscular, aerosol) and platforms including multiple *Mycobacterium tuberculosis* (Mtb) protein subunits in adjuvant and/or expressed in adenovirus vectors. The various platforms and routes are designed to enhance immune responses thought to be involved in protection from Mtb. Despite in certain cases (e.g., aerosol) being able to enhance lung T cell cytokine responses, none of the boosting regimens showed any improved protection over standard intradermal BCG vaccination. This study highlights the formidable challenges facing attempts to elicit robust pulmonary protection against pulmonary Mtb infection.

## Introduction

An estimated one billion people have succumbed to tuberculosis (TB) over the past two centuries.^1^ Currently, *Mycobacterium tuberculosis* (Mtb) infection causes ~1.6 million deaths per year. Bacille Calmette–Guérin (BCG) is the only approved vaccine against TB.^2,3^ BCG given at birth by intradermal (ID) immunization is effective at protecting infants from the systemic manifestations of TB; however, it has variable protective efficacy of 0–80% in adolescents and adults from pulmonary infection and disease, which is the major cause of transmission and death.^1,4^ Thus, vaccine strategies that limit Mtb infection and subsequent disease are urgently needed.^5^ Since BCG confers protection in infants against extrapulmonary manifestations of TB and has partial efficacy against pulmonary infection in adolescents and adults,^4^ the replacement of BCG in newborns is unlikely. Therefore, we tested different prime-boost vaccine regimens to determine whether the efficacy of BCG could be enhanced against pulmonary Mtb infection.

Heterologous prime-boost immunization is a well-established vaccine approach to improve the immunologic responses and protection compared with either vaccine modality alone in mouse, nonhuman primate (NHP), and human studies against infectious diseases.^6^ As BCG is the current standard of care in humans for protection against primary TB, it provides a benchmark against which new boosting regimens can be tested. For boosting following BCG, we assessed two different vaccine platforms and routes. First, we evaluated two protein subunit vaccines, M72 (fusion of Mtb proteins Rv0125 and Rv1196) and H56 (fusion of Mtb proteins ESAT-6, Ag85B, and Rv2660c) with different adjuvants. M72 has been shown to elicit Th1 immunity and some level of protection in mice,^7,8^ guinea pigs,^9^ rabbits,^10^ and cynomolgus macaques.^11^ M72 administered by the IM route with the adjuvant AS01_E_ induces Th1 responses in humans.^12^ H56 provided some level of protection in both pre- and post-Mtb exposure mouse models,^13^ and protected latently infected cynomolgus macaques from anti-TNF induced reactivation.^14^ Boosting BCG with H56 and H4 (a similar vaccine using TB10.4 and Ag85B) administered by IM route with adjuvants increased survival of cynomolgus macaques challenged with high-dose Mtb, compared with BCG alone.^14,15^ H56 administered with CAF01, a liposomal adjuvant designed to elicit Th1 and Th17 immunity,^16^ is thought to mediate protection through long-lived central memory CD4 T cells,^17,18^ and by generating CD4 T cells that express lung homing markers and efficiently migrate to that site.^19^ H56 and the adjuvant IC-31, which activates through TLR 9, showed increased Th1 and antibody responses to the vaccine antigens in humans.^20^ While protein and adjuvant vaccines administered by the conventional IM route can induce circulating CD4 T-cell responses, they may be limited for inducing higher frequency responses in the lung, the site of infection, and for generating CD8 T-cell responses.

Therefore, to address the issues of generating CD4 and CD8 T-cell responses in blood and lung tissue, the second boosting approach we evaluated was to administer a viral-vectored vaccine by the aerosol (AE) and intramuscular (IM) routes. Viral vaccine vectors are highly efficient for inducing T-cell responses in humans to a variety of proteins specific to HIV, Ebola, Malaria, and TB.^21,22^ Indeed, if CD8 T cells have a role in mediating protection, viral vectors are preferable to protein-based vaccines due to the limited ability of the latter approach to induce such responses in humans. Aerosol (AE) immunization is based on the premise that a major hurdle of any vaccination strategy in preventing pulmonary Mtb infection and/or TB disease is the ability to generate a high frequency of antigen-specific tissue resident T cells in the lung to mediate immediate effector function upon TB challenge. Successful elicitation of tissue resident memory T cells (Trm) in the lung is related to both the vaccine formulation and, more importantly, the route of delivery. Indeed, studies in mice and Guinea pigs demonstrate enhanced protection by increasing the numbers of Mtb-specific T cells in the lung through T-cell transfer or mucosal vaccination.^23–25^ In macaques, mucosal delivery of BCG or attenuated Mtb strains increases protection against TB compared with standard BCG ID administration.^26–29^

To generate a high frequency of lung Trm, we used AE delivery of replication-defective recombinant adenoviruses (Ad). We previously demonstrated that AE delivery of an Ad35 expressing Ag85A/B and TB10.4 (Aeras-402) generated robust and durable T-cell responses against the encoded Mtb antigens in the airways of BCG-primed rhesus macaques; however, such responses, did not confer protection against a high-dose (275 CFU) Mtb challenge.^30^ The failure of Aeras-402 to mediate protection alone or as a boost following BCG might be attributed to multiple factors: first, the high-dose challenge with >250 CFU of Mtb might have precluded protection in this highly susceptible rhesus model; second, while Ad35 was selected because of its low seroprevalence in humans, it is far less immunogenic compared with other Ad vectors, such as Ad5^31^; third, the Mtb antigens expressed in Aeras-402 may have been too limited; fourth, the innate or adaptive response in bronchoalveolar lavage (BAL) elicited by AE-delivered Aeras-402 may have altered the lung microenvironment which was detrimental to protection. To address many of these factors and despite its high seroprevalence in humans, here we evaluated Ad5 as a proof of principle, one of the most potent serotypes for inducing T-cell responses, expressing multiple different Mtb antigens, delivered by AE and IM in order to generate lung-resident immunity and a circulating reservoir of memory T cells, and challenged animals with a low-dose Mtb.

The study presented here provides a comprehensive immunologic and protective efficacy analysis using PET/CT and extensive pathologic and microbiologic analysis following vaccination and challenge in the rhesus macaque model of Mtb infection. Overall, the results from this study highlight the limited ability of protein and Ad vaccines to enhance the protection to primary Mtb infection in a highly susceptible NHP model compared with BCG alone.

## Results

### Study design

**Fig. 1:**
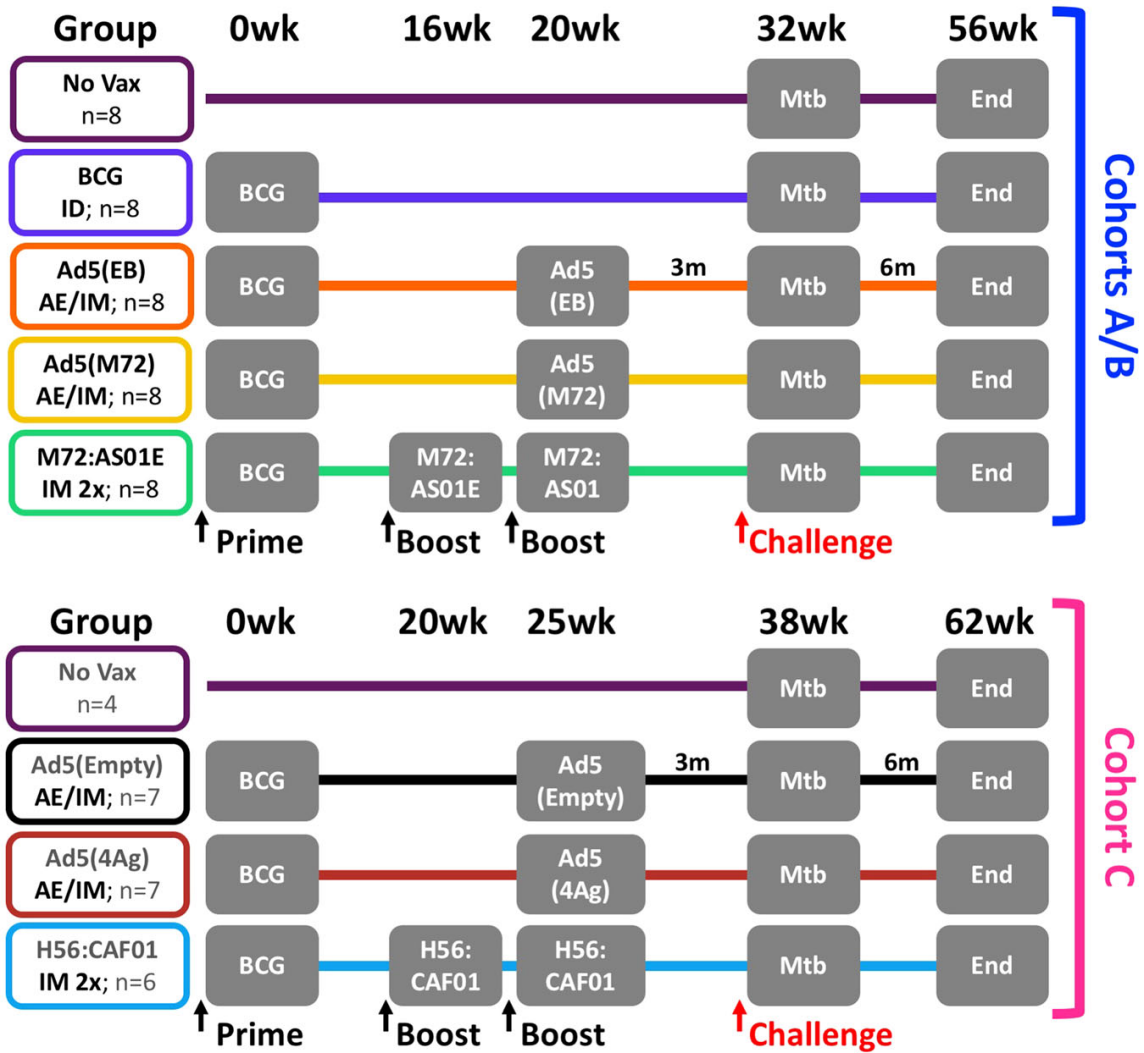
Study design. A total of 64 rhesus macaques were enrolled into three challenge cohorts (A/B, *n* = 20 each; C, *n* = 24) and eight experimental groups: unvaccinated (**No Vax**, *n* = 8 in Cohort A/B and *n* = 4 in Cohort C presented separately because of differences in sampling schedules between cohorts); BCG-primed (**BCG**, *n* = 8); BCG-primed and boosted IM/AE 20 or 25 weeks later with either an empty Ad5 vector [**Ad5(Empty)**, *n* = 7], Ad5 expressing ESAT-6 and Ag85B [**Ad5(EB)**, *n* = 8]; Ad5 expressing M72 [**Ad5(M72)**, *n* = 8]; or Ad5 expressing ESAT-6, Rv1733, Rv2626, and RpfD [**Ad5(4Ag)**, *n* = 7]; BCG-primed and boosted IM twice (4 or 5 weeks apart) 16–25 weeks later with either M72 protein or H56 protein in adjuvant (**M72/AS01**_**E**_, *n* = 8 or **H56:CAF01**, *n* = 6). Three months after boosting (32–38 weeks after BCG), macaques were challenged with a low dose of Mtb Erdman (8–16 CFU) and monitored for 6 months post challenge (study endpoint)

As outlined in Fig. 1, 64 Chinese-origin rhesus macaques were distributed into three sequential challenge cohorts (A, B, and C) and eight study groups: unvaccinated macaques (No Vax, *n* = 12); macaques vaccinated intradermally (ID) with the standard human dose of BCG (BCG, *n* = 8); macaques primed with ID BCG and then boosted AE and IM once at 20 (Cohorts A and B) or 25 weeks later (Cohort C) with an Ad5 vector encoding mycobacterial antigens ESAT-6 and Ag85B [Ad5(EB), *n* = 8], M72 [Ad5(M72), *n* = 8], ESAT-6, Rv1733, Rv2626, and RpfD [Ad5(4Ag), *n* = 7], or no antigen [Ad5(Empty), *n* = 7]; macaques vaccinated with ID BCG and then boosted IM twice, 16–25 weeks later, with either M72 protein (Mtb32A and Mtb39A) plus AS01_E_ adjuvant (M72/AS01_E_, *n* = 8) or H56 protein (ESAT-6, Ag85B, and Rv2660c) in CAF01 adjuvant (H56:CAF01, *n* = 6). Separating animals into three challenge cohorts was necessary due to limitations on numbers of animals that can be concurrently housed and followed under BSL3 conditions. Within each challenge cohort, animals were randomized into comparable groups based on gender, age, and baseline (pre-vaccination) PPD-stimulated responses in BAL, with unvaccinated control animals in each cohort. Three months after the final boost, and ~32 or 38 weeks (Cohorts A/B or C, respectively) after BCG immunization, macaques were challenged via bronchoscope with a low dose (8–16 CFU) of Mtb Erdman. TB disease in each animal was monitored until the humane or pre-defined study endpoint (6 months post-challenge) was met.

### Cellular immune responses in the BAL following BCG-priming and boosting with protein subunit or Ad5 vaccines

**Fig. 2:**
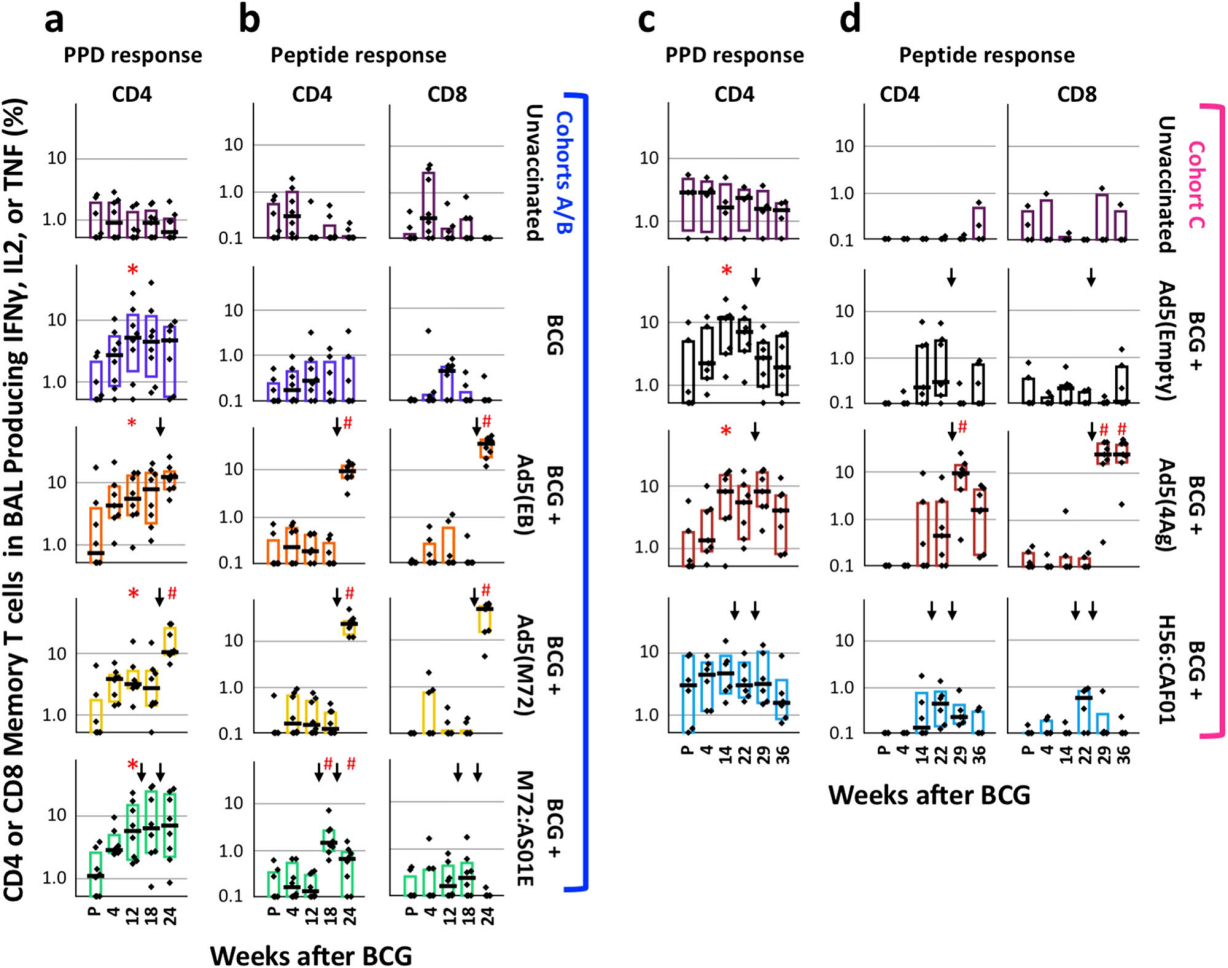
Cellular immune responses in BAL after vaccination. The frequency of memory CD4 or CD8 T cells in the BAL producing any combination of IFNγ, IL2, or TNF in response to PPD (**a**, **c**, CD4 only) or to peptides corresponding to specific antigens delivered in the boost (**b**, **d**) at the indicated weeks before and after BCG immunization. Cytokines were gated as in Fig. S2 and cytokine frequencies are shown on a log_10_ scale. Black arrows indicate the time(s) of boosting. Dots represent individual animals, bars show the interquartile range with the median response indicated. *p ≤ 0.05 compared with pre-vaccination (P) within the same group; ^#^*p* ≤ 0.05 compared with the most recent time point prior to boosting within the same vaccine group using two-tailed Student *t* test

A major aim of the study was to determine whether specific mycobacterial proteins and adjuvant subunit vaccines administered by standard IM immunization or Ad5(TB) vaccines administered by AE and IM immunization could enhance immune responses in the blood and lung following ID BCG priming and enhance protection against TB. To assess changes in the immune responses in the lung after immunization, the number of total T cells (Supplementary Fig. 1) as well as the frequency of antigen-specific T-cell responses was assessed in the BAL after vaccination by multiparameter flow cytometry (Fig. 2; Supplementary Fig. 2). ID BCG priming with or without IM protein plus adjuvant boosting did not alter the number of the total T cells in the BAL (Supplementary Fig. 1a, c). By contrast, administration of all Ad5 vectors via AE/IM increased numbers of CD4 (*p* < 0.02) and CD8 T (*p* < 0.01) cells in the BAL (Supplementary Fig. 1a, c, bar graphs), consistent with prior studies using AE delivery of other Ad5 vaccines.^32^ Of note, Ad5 immunization preferentially increased CD8 T-cell responses, dramatically altering the proportion of CD4 and CD8 T cells in the BAL (Supplementary Fig. 1b, d, pie charts, *p* < 0.006 for 3 of the 4 Ad(TB) vaccines, Ad5(4Ag) is not significantly different from pre-vaccination).

The magnitude of the antigen-specific T-cell response in the BAL after vaccination was measured as the frequency of memory CD4 or CD8 T cells producing any combination of the Th1 cytokines IFNγ, IL2, or TNF in response to PPD (CD4 only, Fig. 2a, c) or peptide (Fig. 2b, d) stimulation. Consistent with prior studies in NHP,^30,33^ ID BCG-immunized macaques generated PPD-specific CD4 T cells in the BAL, measurable 4 weeks after vaccination and peaking at 12–14 weeks. There was limited boosting of these BCG-primed PPD-specific CD4 T-cell responses in the BAL following administration of any of the vaccines, with the exception of AE/IM-delivered Ad5(M72) (*p* < 0.01). In contrast, 4 weeks after boosting with Ad5(EB), Ad5(M72), or Ad5(4Ag) by AE/IM, peptide-specific T-cell responses were increased (*p* < 0.025) from pre-boost levels compared with animals that had received a control Ad5(Empty) boost; CD8 T cells were preferentially boosted (~20–40-fold) compared with CD4 T cells (~10–25-fold). Of note, boosting with M72/AS01_E_ increased peptide-specific CD4 T cells in the BAL after the first (*p* < 0.03) and second (*p* < 0.05) boost, compared with pre-boost (Wk 12). For animals in Cohort C, BAL responses were measured twice after Ad5(4Ag) administration (4 and 11 weeks post boost); in this group, peptide-specific CD8 T cells were maintained to a greater extent than peptide- or PPD-specific CD4 T cells up to 2 weeks before challenge (36 weeks).

A protective role for IL-17 in TB has been reported in mice^34,35^ and more recently in rhesus macaques.^29^ The frequency of IL-17-producing CD4 T cells in the BAL was low after ID BCG immunization, and was not increased by boosting with either Ad5(TB) or protein plus adjuvant boosting (Supplementary Fig. 3a). To delineate which CD4 T cells were producing IL-17, we analyzed the proportion of T cells producing IL-17 in combination with IFNγ, IL2, or TNF producing cells in M72:AS01E boosted animals that displayed modestly increased production of IL-17 (Supplementary Fig. 3b). IL-17+ cells were a small proportion of the the total measured CD4 T-cell response to PPD. In addition, all IL-17 was produced by CD4 T cells that were also making either IFNγ, IL2, and TNF or IFNγ and TNF, as similarly reported by Dijkman et al.^29^

The vaccine formulation, regimen, and route of delivery can influence the immune response quality as defined by the composition of cytokine-producing cells and protective capacity of T-cell responses. Thus, we assessed whether boosting with Mtb proteins plus adjuvant or Ad5(TB) vaccines altered the quality of the BCG-primed CD4 T-cell response in BAL (Fig. S4). As there was no difference in the quality of responses across all vaccine cohorts or groups prior to boosting (*p* > 0.06), all BCG-immunized animals were grouped together to display the overall CD4 quality prior to boosting. PPD-restimulated CD4 T cells produce predominantly IFNγ in combination with TNF, with or without IL-2. While boosting with protein/adjuvant vaccines or Ad5(empty) vaccines did not alter this cytokine profile (*p* ≥ 0.09), boosting with any of the Ad5(TB) vectors elicited a much larger proportion of IFNγ-single positive cells following PPD- or peptide stimulation. Of note, boosting with M72/AS01_E_ maintained a largely multifunctional PPD- and peptide-specific CD4 response that differed from the Ad5(M72)-boosted animals (*p* < 0.005), underscoring the influence that vaccine formulation can have on T-cell quality.

### Cellular immune responses in PBMCs following BCG-priming and boosting with protein subunit or Ad5 vaccines

**Fig. 3:**
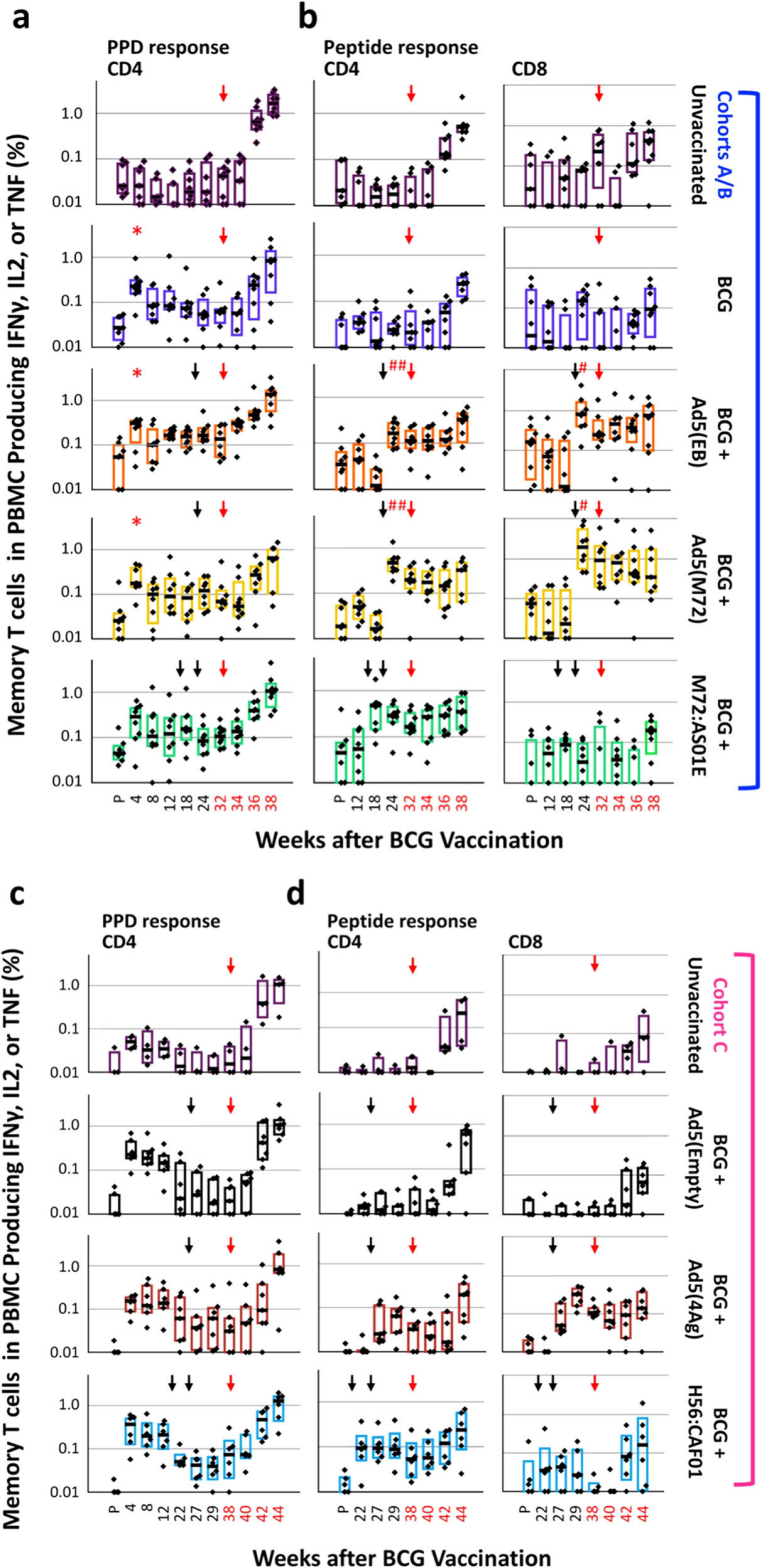
Cellular immune responses in PBMC after vaccination and challenge. The frequency of memory CD4 or CD8 T cells in PBMC producing any combination of IFNγ, IL2, or TNF in response to PPD (**a**, **c**, CD4 only) or to peptides corresponding to antigens delivered in the boost (**b**, **d**) at the indicated weeks before and after immunization and after Mtb challenge (red numbers) is shown for each experimental group (Cohorts A/B, **a**, **b**; Cohort C, **c**, **d**). Black and red arrows indicate the time of boosting and challenge, respectively. Frequencies are shown on a log_10_ scale; dots represent individual animals, bars show the interquartile range with the median response indicated. *p ≤ 0.05 compared to pre-vaccination (P) within the same group; ^#^*p* ≤ 0.05 compared with the most recent time point prior to boosting within the same vaccine group using a two-tailed Student *t* test

Systemic PPD-specific T-cell responses were assessed in PBMCs following ID BCG priming and IM protein/adjuvant or AE/IM Ad5 boosting (Fig. 3). All animals that received ID BCG generated measurable frequencies (ranging from ~0.01–0.05%) of PPD-specific IFNγ, IL2, or TNF producing CD4 T cells by 4 weeks post-priming, with frequencies of ~0.01% at the time of boosting (Fig. 3a, c). There was limited boosting with either the protein/adjuvant or rAd vaccines using PPD for restimulation. By contrast, there were 5–10-fold increases in the antigen peptide-specific CD4 responses following M72/AS01_E_ or H56/CAF01 protein or Ad5(EB), Ad5(M72), or Ad5(4Ag) IM/AE boosting (Fig. 3b, d). PPD-elicited IL-17 production was ~10-fold lower compared with IFNγ, IL2, or TNF, but detectable in some BCG-immunized animals; however, no increase in IL-17 was detected after boosting with any vaccine in response to PPD or peptide stimulation (Supplementary Fig. 3c). Only rAd5-boosted animals showed increases in antigen-specific CD8 T-cell responses (Fig. 3b, d). The maintenance of such responses after boosting until the time of challenge (red arrow) is likely due to systemic (IM) administration of the protein/adjuvant and Ad5 vectors, since we previously demonstrated that delivery of Ad vaccines by AE alone results in only a transient response in blood T-cell responses^30,36^

### BCG vaccination and boosting does not improve mortality after Mtb infection

**Fig. 4:**
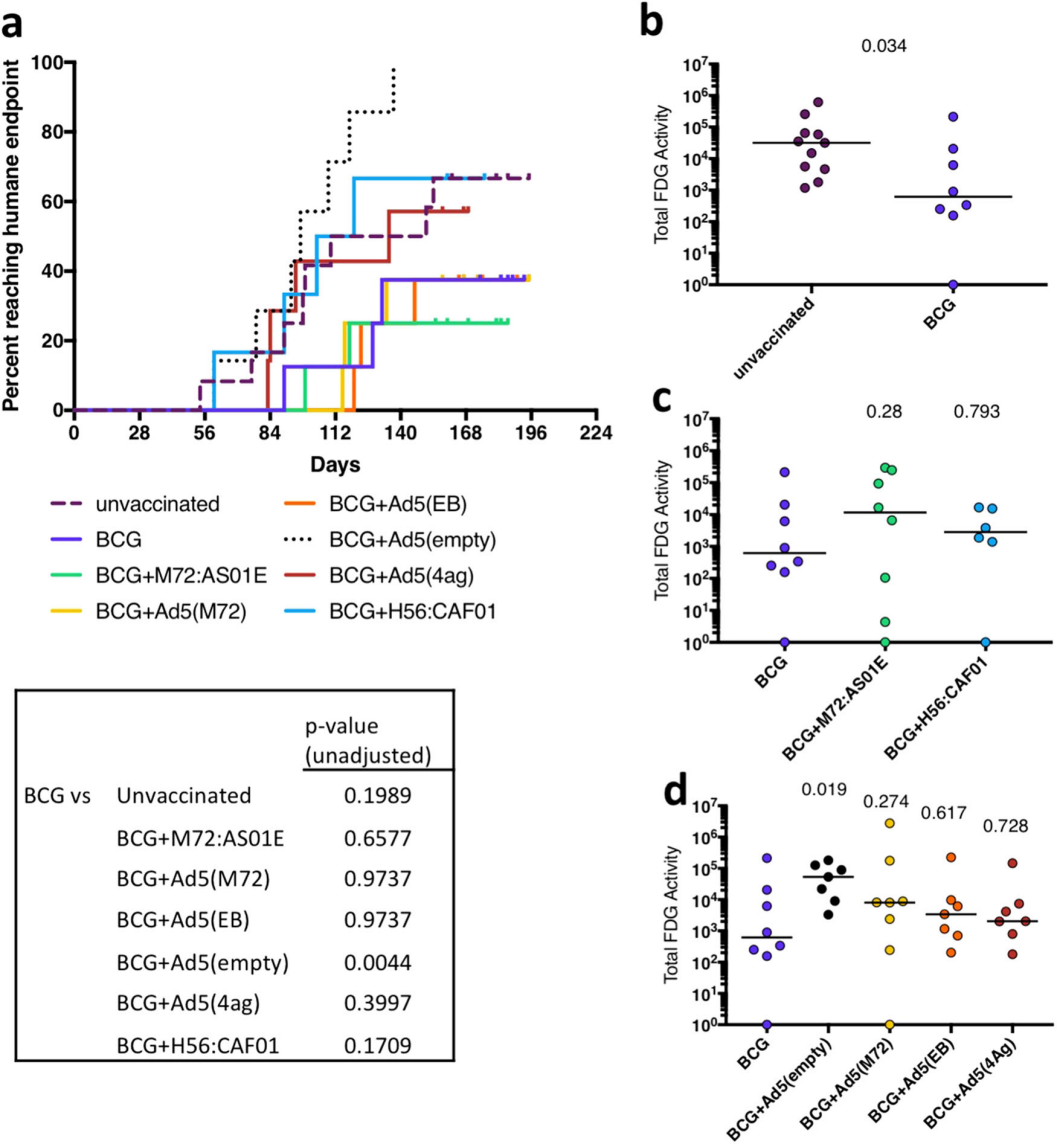
Outcome of Mtb infection in vaccinated macaques. **a** Macaques were monitored clinically for disease, and necropsied if humane endpoints were reached prior to the predetermined endpoint of the study (6 months post-challenge). The fraction of animals reaching humane endpoint are shown on the curve. The survival curve of each treatment group was compared against BCG using the log-rank test and the unadjusted *p*-values are reported. The total FDG activity in the lungs, as a marker of inflammation, was quantified. BCG alone was used as the comparator group for all analyses. **b** Unvaccinated macaques compared with BCG-vaccinated macaques; **c** macaques vaccinated with BCG and boosted with protein plus adjuvant compared to BCG alone; **d** macaques vaccinated with BCG and boosted rAd5(TB) or Ad5(empty) compared with BCG alone. A Kruskal–Wallis test was performed and uncorrected Dunn’s test *p*-values are reported in (**b**, **c**, **d**). Dots represent individual animals and lines represent medians in (**b**, **c**, **d**)

Rhesus macaques are highly susceptible to tuberculosis, even following low-dose challenge,^37^ as was done in this study. The pre-defined endpoint of the study was 6 months post infection, at which time animals were to be necropsied for assessment of Mtb bacterial burden, granuloma extent, and pathology. Any animals reaching humane endpoints using pre-defined clinical criteria (see Materials and Methods and Supplementary Table 1 for individual macaque data) were necropsied earlier. Comparing each group to BCG-vaccinated macaques, only the Ad5(empty) boosted macaques had an increase in fraction of animals reaching humane endpoint (*p* = 0.0044, log-rank test) (Fig. 4a).

### Assessing vaccine efficacy using PET CT imaging

^18^F-fluorodeoxyglucose (FDG) PET CT imaging in macaques has been used as a quantitative noninvasive assessment for determining infection establishment, disease progression, dissemination of infection and response to vaccines or drug treatment.^37–41^ The total FDG activity in the lungs is a measure of lung inflammation, and correlates with the total thoracic (lung + thoracic lymph node) bacterial burden in animals infected with Mtb.^37^ Here, serial PET CT imaging was used to assess progression of infection and extent of disease in all macaques. For final comparisons, the total lung FDG activity in the pre-necropsy scan was used. Vaccination with BCG reduced the total lung inflammation (FDG activity) compared with unvaccinated macaques (*p* = 0.034), although there was a wide range in both groups (Fig. 4b). However, boosting BCG with M72 protein in AS01_E_ or H56 protein in CAF01 did not alter lung inflammation compared with BCG alone (Fig. 4c), again with a wide range among animals in each group. Similarly, boosting BCG with Ad5 vectors expressing various Mtb proteins did not alter lung inflammation compared with BCG alone (Fig. 4d). Of note, there was an increase in lung inflammation in animals boosted with the control (empty) Ad5 vector administered after BCG compared with BCG alone (*p* = 0.019) (Fig. 4d). Overall, serial PET CT scans show early increases in FDG activity (inflammation) in the lungs in nearly all macaques, with variability in FDG activity over time and among animals in each group (Supplementary Fig. 5).

### Bacterial burden at necropsy

**Fig. 5:**
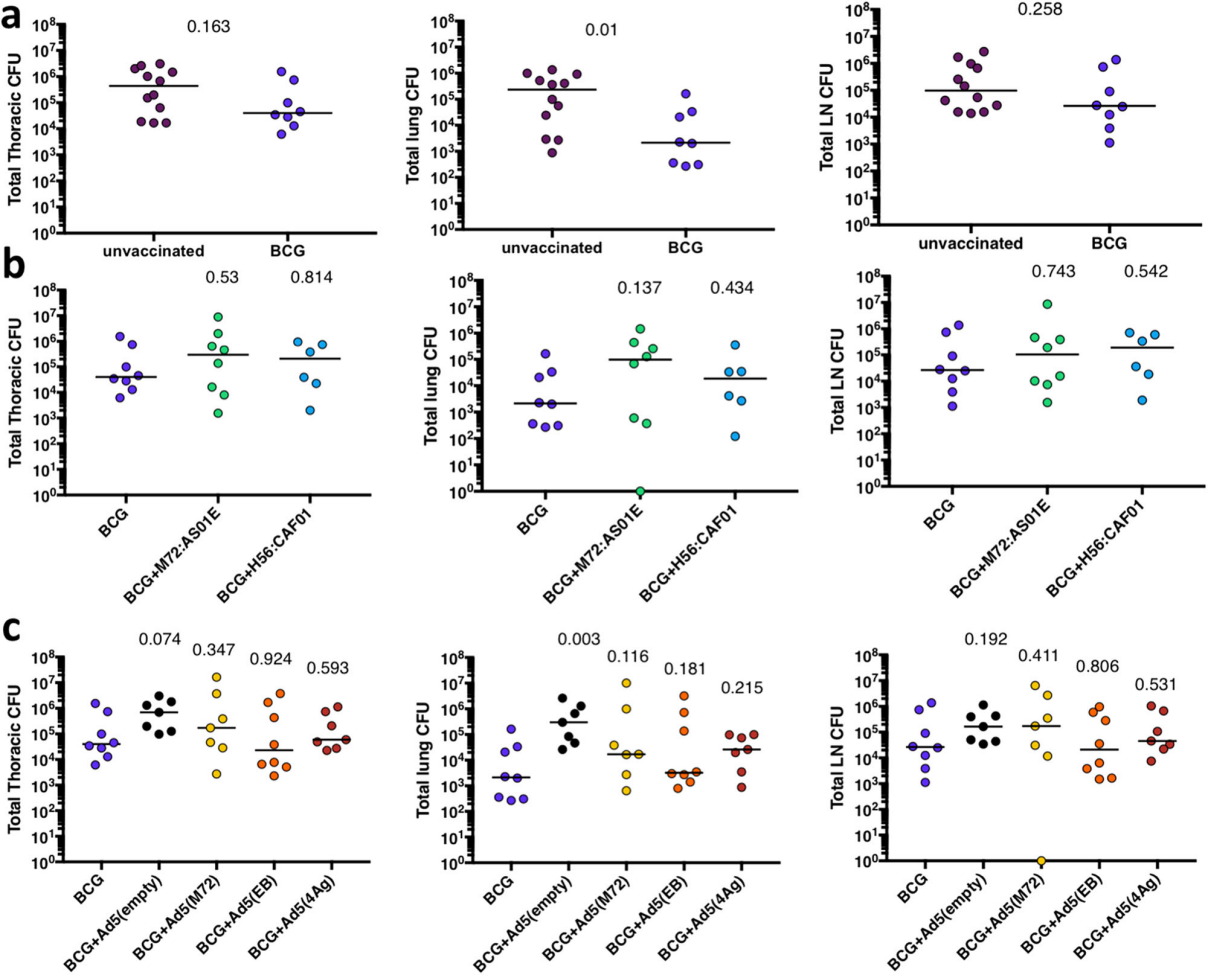
Bacterial burden at necropsy. The total thoracic (lung + lymph node) bacterial burden (left panels), the total lung bacterial burden (middle panels), and the total thoracic lymph node bacterial burden (right panels) at necropsy were compared for BCG alone vs. unvaccinated macaques (**a**), BCG alone vs. BCG boosted by protein plus adjuvant (**b**), and BCG alone vs BCG boosted with rAd(TB) or Ad(empty) vectors (**c**). A Kruskal–Wallis test was performed and uncorrected Dunn’s test *p*-values are reported. Dots represent individual animals and lines represent medians

Detailed necropsies were performed on each macaque at humane or pre-defined time points (Supplementary Table 1). Using a previously established gross pathology scoring system used to assess disease severity in NHP,^37^ there were no differences between unvaccinated or any of the BCG primed or prime-boosted macaques (Supplementary Fig. 6a). The total Mtb bacterial burden was assessed as previously described.^37,42^ All granulomas, thoracic, and peripheral lymph nodes were individually recovered using the final PET CT scan to specifically localize lesions, and plated individually for bacterial burden. In addition, random sampling of uninvolved lung tissue (random tissue pieces equally approximately half of each lobe), spleen (1–3 granulomas if present, and ~1/3 of the remainder of spleen), and liver (1–3 granulomas if present, and ~half of three different liver lobes) was performed to assess bacterial burden in these tissues, as previously described.^37,42^ The total thoracic bacterial burden was determined from the combination of all lung and thoracic lymph node samples. Although the total thoracic bacterial burden was not different between unvaccinated and BCG-vaccinated macaques, analysis of the lung and lymph node components separately indicate that lung bacterial burden was reduced by BCG vaccination alone (*p* = 0.01), while thoracic lymph node bacterial burden was similar to the unvaccinated controls (Fig. 5a). Boosting BCG with the protein and adjuvant vaccines (Fig. 5b) or Ad5 vectors (Fig. 5c) did not reduce the total Mtb bacterial burden compared with BCG alone; lung and lymph node bacterial burdens in BCG boosted animals were also similar to BCG alone. However, boosting with empty Ad5 vector increased the total thoracic bacterial burden compared with BCG alone (*p* = 0.074) (Fig. 5c), as was suggested by the PET CT data (Fig. 4d). Overall, although ID BCG vaccination alone provides limited protection in rhesus macaques, particularly with respect to lung bacterial burden, boosting with proteins in adjuvant or proteins expressed by Ad5 vectors did not improve protection against TB as measured by live imaging, pathology, bacterial burden, or mortality.

### Extrapulmonary disease

Dissemination of Mtb infection is a sign of poorly controlled disease. Thus, the extent of extrapulmonary disease observed at necropsy (i.e., numbers and size of granulomas in the liver, spleen, or other extrapulmonary organs) and the culture positivity of a random sampling of extrapulmonary lesions from each organ was determined.^37^ Extrapulmonary disease is more common in rhesus macaques compared with cynomolgus macaques,^37^ so this model provides a useful system for assessing prevention of dissemination. As expected, ID BCG alone reduced the extent of extrapulmonary disease compared with unvaccinated controls consistent with its effect on reducing disseminated disease in human infants (*p* = 0.036) (Supplementary Fig. 6b). However, neither the proteins plus adjuvants or the Ad5(TB) vaccines showed improvement compared with ID BCG alone in limiting extrapulmonary disease) (Supplementary Fig. 6b).

### PET CT imaging correlates with outcome of infection in vaccinated animals

**Fig. 6:**
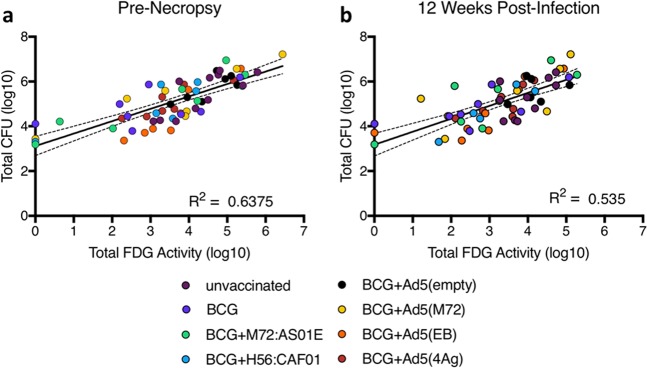
PET imaging correlates with bacterial burden. The total lung FDG activity correlates with total thoracic bacterial burden (lung + lymph node) at necropsy (**a**). The total lung FDG activity at 12 weeks post-infection correlates with bacterial burden at necropsy weeks to months later (**b**). The results of simple linear regression indicate that there is a positive association between the total CFU and the total lung inflammation at necropsy (R^2^ = 0.6375, F = 103.8, *p* < 0.0001) and at 12 weeks post infection (R^2^ = 0.5350, F = 65.6, *p* < .0001). Dots represent individual animals and dotted lines represent 95% confidence intervals

We previously reported a correlation between FDG activity in the lungs and the total thoracic bacterial burden at the time of necropsy in unvaccinated macaques (rhesus and cynomolgus species).^37^ Here, we assessed whether there was a correlation between FDG activity in lungs and the total thoracic bacterial burden in vaccinated monkeys following Mtb challenge (Fig. 6). At necropsy, the total FDG activity in lungs was positively correlated with the total bacterial burden (*r* = 0.64, *p* < 10^−4^), confirming that live PET CT imaging is a useful outcome measure in a vaccination study following infection (Fig. 6a). To determine whether outcome of infection in vaccinated macaques can be predicted at an earlier time point post-challenge, we assessed the relationship between FDG activity in lungs at 12 weeks post infection with thoracic bacterial burden at necropsy and found that lung FDG activity at 12 weeks positively correlated with necropsy bacterial burden (*r* = 0.54, *p* < 10^−4^) (Fig. 6b).

### Comparing vaccines across platforms

The design of this study provides the opportunity to compare Mtb infection outcomes between macaques receiving vaccines by two different platforms and routes. Moreover, for the M72 antigen, we were able to compare how a protein and adjuvant and Ad5 vector for the same antigen, albeit delivered by different routes, influences immunity and protection. For the other vaccines used, the antigens were similar but not identical: rAd5 expressed an ESAT-6/Ag85B fusion protein, while the H56 fusion protein delivered with adjuvant consists of Ag85b/ESAT-6/Rv2660c. We did not find differences in protection, as measured by lung FDG activity, bacterial burden or extrapulmonary disease, between the same or similar antigens administered as protein fusions in adjuvant or expressed in recombinant Ad5 vectors (Supplementary Fig. 7).

### No correlation of peripheral or airway T-cell responses with infection outcome

Although no vaccine group showed increased protection compared with BCG alone, there were individual macaques in several vaccine groups that had less disease than others. We investigated whether the individual blood or BAL T-cell responses were correlated with outcome across all macaques in the study. The peak BAL CD4 or CD8 T-cell response to PPD stimulation (or vaccine peptide stimulation) for each animal was tested for correlation against the total thoracic bacterial burden, and no correlation was found (Supplementary Fig. 8). Similar analysis was performed on peak CD4 or CD8 T-cell responses in PBMC and again there was no correlation with the total thoracic bacterial burden) (Supplementary Fig. 9). Thus, inducing a high frequency of mycobacterial-responsive T cells in the airways or blood following boosting of BCG in this model is insufficient to provide improved protection over BCG alone against infection or disease.

### Vaccine-induced T-cell responses in PBMC are not boosted after Mtb Challenge

An important aspect of any vaccine-elicited cellular immune response is its capacity to respond to infectious challenge more rapidly than a primary response. We therefore investigated the anamnestic CD4 and CD8 T-cell responses in PBMCs at 2, 4, and 6 weeks after Mtb challenge (red arrow, Fig. 3). All vaccinated and unvaccinated animals had frequencies of PPD-specific T cells that increased in the PBMCs after challenge consistent with the increase in mycobacterial burden (Fig. 3a). However, such responses were not higher or more rapid in vaccinated animals compared with unvaccinated controls. Similarly, vaccine-elicited responses to the specific peptides from each vaccine did not increase immediately following challenge. (Fig. 3b). These data show that pre-existing vaccine-elicited responses in the blood were not appreciably boosted upon Mtb challenge. Of note, we cannot rule out that anamnestic responses occurred in the lung (which were not sampled post challenge).

### Cellular kinetics in BAL after AE immunization

We previously showed that AE administration of Aeras-402 (Ad35 encoding Ag85A/B and TB10.4) induced robust T-cell responses in BAL of NHP, but provided limited protection following a high-dose challenge. Here, we confirm these findings with a more immunogenic vector (Ad5) encoding additional Mtb antigens in the context of a low-dose (8–16 CFU) challenge. Given that potent cellular immune responses in the BAL after AE Ad5(TB) immunization did not enhance protection in animals and the observation that AE Ad5(empty) may have undermined the protective effect of BCG priming, we investigated early innate immune activation in the BAL after Ad5 administration. Such anti-viral (Type I IFN) innate responses have been associated with disease progression and poor outcome in both humans and in mouse models.^43–45^ Nine animals were administered saline (*n* = 3) or Ad5(EB) (*n* = 6) by the AE route (only) and changes in BAL cellular composition (Supplementary Fig. 10) and innate cytokine milieu (Supplementary Fig. 11) were examined over one month. The composition of the BAL was altered at 14 and 28 days after Ad5(EB), due to an increase in the proportion of CD8 T cells (Supplementary Fig. 10A) that expressed markers of lung tissue residency (CD69 and CD103) (Supplementary Fig. 10B). Assessment of innate and inflammatory cytokines including IFNα, IFNβ, IP-10, IL-1β, IL-1Ra, IL-10, and IL-12 at all time points in plasma and BAL fluid, however, failed to show differences between the saline and Ad5(EB) animals that would provide insight into a detrimental innate environment following AE Ad5 administration (Supplementary Fig. 11).

## Discussion

The goal of this study was to assess whether administration of protein subunit or viral vector vaccines after BCG priming would boost immunity and confer additional protection against Mtb challenge in a nonhuman primate model. We hypothesized that inducing a high frequency of Mtb-specific T cells at the portal of infection (the lung) should provide protection against TB challenge. Rhesus macaques were chosen due to their increased susceptibility to developing active TB disease compared with cynomolgus macaques, providing a stringent challenge model.^37^

Here, we tested two different vaccine platforms for boosting (protein plus adjuvant and Ad5 vectors), various combinations of eight Mtb antigens (some of which have been tested in limited clinical trials), and parenteral versus pulmonary delivery. We used multiple outcome measures to define protection in this model system, including PET CT imaging, pathology scoring, and accurate bacterial burden determination. BCG provided limited protection compared with unvaccinated macaques, particularly in reducing lung inflammation and overall lung bacterial burden, consistent with prior studies.^27,28,33^ However, despite robust T-cell responses in the airways of macaques, in which BCG was boosted with AE/IM Ad5(M72), Ad5 (EB), or Ad5(4Ag), there was no additional protection compared to BCG. Similarly, peripheral (IM) boosting with protein antigens (in adjuvant) that were matched (M72) or similar (H56) to antigens encoded by the Ad5 vectors [Ad5(M72) or Ad5(EB)] failed to enhance the limited protection conferred by BCG in this low-dose challenge model. A previous study showed no increase in survival time, but reduced CFU in cynomolgus macaques, vaccinated with BCG and M72 in AS02A compared to BCG alone following Mtb high-dose challenge.^11^ Similarly, BCG boosted by H56 in CAF01 also provided modest protection against high-dose Mtb challenge in cynomolgus macaques, and protection against reactivation of latent infection following low-dose Mtb challenge.^14^ These results highlight that even in cynomolgus macaques, which are more resistant to TB disease than rhesus, there is modest efficacy of subunit vaccines against primary infection. Together, these data raise the critical question of how predictive the various NHP models are for assessing protection against primary infection and disease in humans.

With regard to the route of vaccine administration, we compared AE and IM immunization to determine whether lung-targeted vaccination was more efficacious in boosting BCG-elicited protection compared with vaccines administered by the standard peripheral route. Although AE/IM delivery of each Ad5-TB vector generated CD4 and CD8 T cells against its corresponding Mtb antigens in both the BAL and blood, none of the Ad5-TB vaccines enhanced BCG-induced protection. Thus, generating high levels of CD4 and/or CD8 T cells, even locally in the lung, is not sufficient to protect against TB disease. These data are in contrast to experiments in mice showing that mucosal immunization with Ad5 expressing Ag85A (AdAg85A) is more protective than IM AdAg85A due to greater accumulation and retention of CD4 and CD8 T cells in the airways.^25^ However, when this construct was tested by AE immunization with AdAg85A in BCG-primed NHPs, there was no additional protection compared with BCG alone, and no differences in protection between IM and AE administered AdAg85A.^46^ An important aspect that also differs between mouse and our NHP studies is the method by which the vaccines are administered by AE route. Here, we use a PARI device that administers the vaccine by aerosol in 4 µm droplets. Mouse studies use intranasal administration. However, it is possible that in this study, administering the Ad5-vectored vaccines by both IM and AE routes together could have impaired protection given by either route alone.

A notable finding was that Ad5(TB) vectors did not provide additional protection beyond BCG alone, despite inducing potent T-cell responses to multiple Mtb antigens. Indeed, AE/IM administration of the Ad5(empty) vector to BCG-vaccinated animals resulted in worse lung disease, compared with BCG alone. This finding suggests that the Ad5 vector alone may alter the lung to promote susceptibility, and this may be offset by the increase in T-cell responses elicited when Mtb antigens are included in the Ad5 vectors. The host immune response to viral infection can have a major influence on immunity and protection to concomitant or subsequent infection. Specifically, anti-viral type I IFN production has been shown in TB patients and in some mouse models to correlate with, or predispose to, worse TB disease.^43–45,47,48^ In this regard, vaccine delivery platforms that promote strong innate immune activation and type I interferon in the lung may limit vaccine efficacy. Ad5 is one of the most immunogenic of serotypes of adenoviral vaccine vectors for inducing T-cell immunity and is consistent with the potent responses observed in this study.^49–52^ The lack of increased protection afforded by such responses prompted us to investigate the innate cellular and cytokine environment in the airways following AE administration of Ad5. We were unable to detect changes in the innate cell subsets, or consistent changes in innate mediators that were measured in BAL fluid in animals receiving saline or Ad5(EB) AE to support a detrimental role of the innate environment in the airway after Ad5 AE vaccination that would limit protection. There remain several possibilities for the failure of Ad5-elicited responses to increase protection. First, Ad5 preferentially increases CD8 T cells compared with CD4 T cells that may have a more critical role in mediating protection against primary infection. Second is that there is insufficient antigenic breadth with the antigens used in these vaccine constructs; the lack of anamnestic responses after challenge suggests that the immunizing antigens were either not present or recognized post challenge, at least in the blood. In a prior study, we also failed to demonstrate protection by Ad-vectored vaccine given by IM or AE alone^30^ in rhesus macaques. Thus, the cumulative data so far in NHP suggest that Ad vectors may be limited for inducing protection against TB, at least by the AE delivery route. Based on the striking protection seen in rhesus macaques following vaccination with CMV encoding several similar Mtb antigens,^33^ these data highlight major differences in how viral vectors mediate protection in this model.

This study was initiated several years ago, coincident with a Phase IIb clinical trial testing M72/AS01_E_ in latently infected adults in Africa. The recent analysis of this clinical trial showed 54% protection against pulmonary TB disease in adult humans that had anti-TB immune responses (IGRA+) at the time of vaccination.^53^ These are the first data to show that the M72/AS01_E_ subunit vaccine, which was also used here, can have a demonstrable protective effect in humans against pulmonary TB disease in subjects with LTBI. Here, we assessed protection against primary infection (acquisition) and disease progression in Mtb-naive macaques. It is possible that M72/AS01_E_ boosting in adult humans with recently established LTBI induces strong recall responses to the initial *M. tuberculosis* infection that protect against subsequent reactivation or possibly reinfection. Indeed, we recently published that concurrent infection with Mtb in cynomolgus macaques provides robust protection against reinfection.^54^ Thus, there may be major differences in how vaccines protect in the setting of concurrent infection versus being IGRA negative. Another potential limitation of our experimental model is that BCG was given ~16–20 weeks before the first boost with protein/adjuvant or 20–25 weeks prior to boosting with Ad5, whereas humans are typically vaccinated with BCG at birth and therefore boosting occurs 10–20 years later. A proximal BCG prime might interfere with immunogenicity or protective capacity of subsequent booster immunization, as suggested by a recent study in which protection afforded by a rhesus CMV TB vaccine was ameliorated by BCG vaccination 6 weeks earlier.^33^

We have previously reported on the utility of PET-CT as a major noninvasive technical advance for assessing Mtb infection and disease in NHPs, with serial imaging to monitor dissemination,^41,55^ assess infection outcome,^37,54–56^ evaluate drug efficacy,^38,39^ and determine risk of reactivation disease.^42^ Here, we show that quantification of lung associated inflammation by PET can identify protected macaques in a vaccine study. The total FDG activity in lungs is correlated with the total thoracic bacterial burden at necropsy in vaccinated animals, and a similar measurement at 12 weeks post infection is a reasonable predictor of bacterial burden weeks to months later. This noninvasive technology is a much less time-consuming method than quantifying true bacterial burdens from macaques at necropsy, and can be considered an alternative quantitative outcome measure in vaccine studies.

In summary, these data show that it is very challenging to enhance the limited protection provided by BCG ID alone by boosting with many of the current subunit vaccine approaches being tested in humans in this highly susceptible NHP model.

## Materials and methods

### Animals

All animal studies were approved by the Animal Care and Use Committees of the Vaccine Research Center, National Institute of Allergy and Infectious Diseases, NIH, of Bioqual, Inc., and of the Institutional Animal Care and Use Committee of the University of Pittsburgh. We have complied with all ethical regulations regarding animal research. Animals were housed and cared for in accordance with local, state, federal, and institute policies in facilities accredited by the American Association for Accreditation of Laboratory Animal Care, under standards established in the Animal Welfare Act and the Guide for the Care and Use of Laboratory Animals. Animals were pair-housed at Bioqual, Inc., throughout the vaccination phase and were monitored for physical health, food consumption, body weight, temperature, complete blood counts, and serum chemistries. All infections were performed at the University of Pittsburgh, and the macaques were housed in a Biosafety Level 3 facility. The bacterial burden and PET CT data from the 12 unvaccinated (naïve) macaques were published previously in a non-vaccine related paper^34^ (also noted in Supplementary Table 1), although these animals were infected and analyzed concurrently with the vaccinated animals from each cohort.

### Vaccination

Sixty-four, 3–5-year-old, male and female Chinese-origin rhesus macaques (*Macaca mulatta*) were used for challenge studies. Animals were randomized into experimental groups based on age, weight, gender, and pre-existing CD4 T-cell responses to PPD in the BAL. Animals with positive plasma neutralization titers for Ad5 were assigned to groups without Ad5 immunization. Animals were immunized at Bioqual, Inc. in three successive cohorts (A/B and C; Cohort A/B included four out of eight animals in each vaccine group), each cohort containing four unvaccinated ‘infection controls’ (No Vax). All other groups received the standard human dose of (2–8 × 10^5^ CFU) BCG Danish strain 1331 (Statens Serum Institute, Copenhagen, Denmark), prepared according to the manufacturer’s instructions for human use, intradermally (ID) in the upper arm in a volume of 0.1 ml, at week 0. Four groups received a single boosting immunization with a replication-defective recombinant human adenovirus serotype 5 (Ad5) vector expressing antigens from BCG, Mtb, both, or neither: Ad5(Empty), control vector; Ad5(EB), encoding ESAT-6 (Rv3875) and Ag85B (Rv1886); Ad5(M72), encoding Mtb32A (Rv0125) and Mtb39A (Rv1196); Ad5(4Ag), encoding ESAT-6, Rv1733, Rv2626, and RpfD (Rv2389c). Ad5(EB) was provided by Sheldon Morris (US FDA). Ad5(empty) and Ad5(M72) were constructed using the AdZ5 vector system (Gavin Wilkonson, Cardiff University).^57^ All Ad5 vaccines were delivered both intramuscularly (IM) in the quadricep and by aerosol (AE) in a volume of 1 ml of saline (each). Animals received a total of 2 × 10^10^ viral particles (vp) of Ad5(EB) or Ad5(M72) at week 20 post-BCG (Cohort A/B), or 4–6 × 10^10^ vp of Ad5(Empty) or Ad5(4Ag) at week 25 post-BCG (Cohort C), with the the total dose divided equally between the IM and AE routes. For AE delivery, the eFlow rapid nebulizer system (Pari Pharma GmbH, Starnberg, Germany) with a pediatric face mask was used to deliver 4 micron particles deep into the lung of an anesthetized macaque, as described.^32^ Two groups of animals received two booster immunizations, 4 or 5 weeks apart with mycobacterial proteins in adjuvant: M72/AS01_E_ (GSK, Rixensart, Belgium; M72: Mtb32A and Mtb39A) or H56:CAF01 (ESAT-6, Ag85B, and Rv2660c) IM in the quadricep. AS01_E_ (GSK, Rixensart, Belgium) is a liposome-based adjuvant system comprising 25 µg of the Toll-like receptor 4 ligand MPL (3-*O*-desacyl-4′-monophosphoryl lipid A), and 25 µg QS-21 (*Quillaja saponaria* Molina, fraction 21. Licensed by GSK from Antigenics LLC, a wholly owned subsidiary of Agenus Inc., DE, USA corporation), per injected dose.

Animals received 10 ug of M72 protein in 0.5 ml AS01_E_ adjuvant^12,58^ at weeks 16 and 20 (Cohort A/B) or 25 µg of H56 protein in 0.3 ml of CAF01 adjuvant^20,59^ at weeks 20 and 25 (Cohort C). Animals were transferred to the University of Pittsburgh 25–30 weeks post-BCG, quarantined for 6 weeks, and transferred to an ABSL-3. Nine adult rhesus macaques were used for the cellular kinetics study; three received saline AE, and six were immunized with 2 × 10^10^ vp of Ad5(EB) AE. To avoid frequent BAL sampling on the same animals, the six Ad5(EB) immunized animals were sampled on an alternating schedule.

### *M. tuberculosis* challenge

Animals were challenged with 8–16 CFU Erdman strain *Mycobacterium tuberculosis* (Mtb) in a volume of 2 ml via bronchoscope. Animals were clinically monitored and examined daily, including appetite, behavior, weight, erythrocyte sedimentation rate (ESR), activity, Mtb growth from gastric aspirate, and coughing. These signs were used as criteria in determining whether an animal qualified as meeting humane endpoint prior to the scheduled study endpoint (6 months post challenge).

### Necropsy, pathology scoring, and bacterial burden

The macaques in this study were euthanized ~6 months (24–28 weeks) after Mtb challenge, or at humane endpoint. All animals were euthanized with an intravenous overdose of sodium pentobarbital (Beuthanasia) and maximally bled. At necropsy, each animal was examined grossly for pathology. Using our published scoring system,^37^ the total amount of pathology was recorded from each lung lobe (number and size of lesions), LN (size and extent of necrosis), and extrapulmonary compartments (number and size of lesions). A pre-necropsy PET CT scan was used as a “map” for identifying and finding each lesion in the lungs. Each individual lesion (including granulomas, consolidations, clusters of granulomas) in the lung and all thoracic LNs identified were excised and mechanically homogenized to create a single-cell suspension. Portions of the suspensions were spread on 7H11 agar (Difco) and incubated at 37 °C with 5% CO_2_ for 3 weeks. CFU were then counted and used to quantify both individual lesion bacterial burden and—when summed together—the total bacterial burden for the animal.^37,60,61^ Extrapulmonary disease score was determined as described in Maiello et al.^37^

### PET CT scans and analysis

PET CT scans were performed using a microPET Focus 220 preclinical PET scanner (Siemens Medical Solutions) and a clinical eight-slice helical CT scanner (Neurological Corp.) as previously described.^37,38,40,56^ ^18^F-fluorodeoxyglucose (FDG), a non-specific marker of inflammation, was utilized as the probe for PET. Serial PET-CT scans were performed between 2 and 24 weeks post-Mtb infection, and before necropsy. OsiriX viewer, an open-source PACS (Picture Archiving and Communication System) workstation and DICOM (Digital Imaging and Communications in Medicine) image viewer, was used for scan analyses as previously described.^56^ Briefly, a region of interest (ROI) was segmented which encompassed all lung tissue on CT and was then transferred to the co-registered PET scan. On the PET scan, all image voxels of FDG-avid pathology (SUV_max_ > 2.3) were selected and summated resulting in a total SUV_max_ value. To account for basal metabolic FDG uptake in the animal, the total SUV_max_ was normalized to resting muscle resulting in a total lung inflammation value. Individual granulomas as seen on CT were counted in each scan to enumerate them longitudinally.

### Flow cytometry

T-cell assays were performed on freshly isolated BAL throughout the pre-challenge phase, but not after challenge. BAL was harvested from anesthetized macaques by collecting three consecutive 20-ml washes with saline. BAL cells were resuspended in warm R10 (RPMI 1640 with 2 mM L-glutamine, 100 U/ml penicillin, 100 µg/ml streptomycin, and 10% heat-inactivated FCS) containing 2 U/ml DNAse (Millipore Sigma), filtered through a 70-µm cell strainer, and rested for 2–4 h before overnight (16 h) stimulation with antigen. Cryopreserved PBMC was batch-analyzed at the end of study. PBMC was isolated from heparinized whole blood separated with Ficoll, washed twice with saline, and frozen in 90% FCS/10% DMSO. PBMC was thawed in a 37 water bath, washed twice in warm R10 containing 50 U/ml Benzonase (Millipore Sigma) and rested overnight in R10 before a 6-h stimulation. Antigen stimulation was performed by culturing BAL cells or PBMCs with 20 µg/ml purified protein derivative (PPD) S-2 (Provided by FDA) or 1 µg/ml (each) of overlapping peptide pools corresponding to each antigen contained in the Ad5 or protein/adjuvant vaccines (i.e., ESAT-6, Ag85B, Mtb32a, Mtb39a, Rv1733, Rv2626, RpfD, Rv2660c). Cells from unvaccinated and BCG-immunized animals were incubated with peptides corresponding to the antigens contained in the Ad5 or protein/adjuvant vaccine groups within the same cohort (i.e., ESAT-6, Ag85B, Mtb32a and Mtb39A for Cohorts A/B). BD GolgiPlug (BD Biosciences) was added at a concentration of 10 µg/ml 2 h after antigens. Immunostaining for flow cytometry included: viability staining using Live/Dead Fixable Aqua Dead Cell Stain (ThermoFisher Scientific); surface staining for CD4 (OKT4, Biolegend #317442, 1:25), CD8 (RPA-T8, Biolegend #301038; 1:40), CD28 (CD28.2, BD Biosciences #555730; 1:10), CD45RA (L48, BD Biosciences #337167; 1:200), CD103 (2G5, Beckman Coulter #IM1856U; 1:25), CCR7 (G043H7, Biolegend #353208; 1:25); intracellular staining of CD3 (SP34.2, BD Biosciences #557757: 1:150), CD69 (TP1.55.3, Beckman Coulter #6607110; 1:25), IFNg (B27, BD Biosciences #554702; 1:150), IL2 (MQ1–17H12, BD Biosciences #554566; 1:150), TNF (Mab11, BD Biosciences #563418; 1:25), IL-17 (N49-653, BD Biosciences #560799; 1:50; except Cohort A BAL). Fixation and permeabilization was performed using BD Cytofix/Cytoperm (BD Biosciences). Sample gating was performed as described;^29^ briefly CD69+ cytokine-producing cells were gated on memory (non-CD28+CD45RA+) CD4+ or CD8+, CD3+ T cells. For cellular kinetics, BAL cells were surface stained to delineate multiple leukocyte populations using the following antibodies (clone and dilution listed if different from above): CD3, CD4 (SK3, BD Biosciences # 564305; 1:40), CD8, CD103, CD69, CD163 (GH1-61, Biolegend #333622; 1:40), CD14 (M5E2, Biolegend #301840; 1:80), CD16 (3G8, BD Biosciences #564653; 1:40), CD20 (2H7, Biolegend #302332; 1:40), CD66abce (TET2, Miltenyi #130-116-668, 1:50), NKG2A (Z199, Beckman Coulter #A60797; 1:80), HLA-DR (TU36, ThermoFisher Scientific #MHLDR18; 1:400), CD11c (3.9, Biolegend #301608; 1:200), CD123 (6H6, Biolegend #306008; 1:160), CD45 (D058-1283, BD Biosciences #563530; 1:40). The data were acquired on a modified BD LSR II and analyzed using FlowJo software (BD). Background subtraction was performed by subtracting the frequency of cytokine production from the identical sample incubated without antigen.

### Luminex and ELISA

Cytokines present in 10-fold concentrated BAL wash fluid (PBS) and plasma were measured using a Milliplex Non-human Primate Cytokine Magnetic Bead Panel cytokine/chemokine 23-analyte multiplex assay (Millipore Sigma), or a monkey IFN beta ELISA kit (My Biosource) according to the manufacturer’s instructions.

### Statistics

Graphics and statistics in Figs. 4, 5, 6, S6, and S7 were created using Prism (Graphpad). In these figures where indicated: comparisons of time to humane endpoint were done using the log-rank test and comparisons of two groups were performed using the uncorrected Dunn’s test. Graphs and statistics in figs. S5, S8, and S9 were created using JMP Pro (SAS), where Spearman’s rho was calculated for pairwise correlations. Most of these data were tested for normality and failed; therefore, non-parametric two-sided tests were utilized. The exception was data in Fig. 6, where a log_10_ (+1) transformation was performed for both variables. Analysis and presentation of flow-cytometric data (Figs. 2, 3, S1, S2, S3, S4, S10 and S11) was performed using Pestle and SPICE (Ver. 5.23; https://exon.niaid.nih.gov/spice/);^62^ statistical analysis was performed using a two-tailed Student *t* test to compare absolute numbers and frequencies, and a permutation test to compare pie charts. One subject (8513) is missing from CFU analysis (the total thoracic, lung, and lymph node) as a result of contamination of bacterial plates. There are two animals with missing PET scan values for the final time point. One of these scans was terminated for the safety of the animal (6914) due to airway compression. The other missing value (7913) was the result of a completely collapsed lung lobe. Descriptive statistics summarized by treatment group for all necropsy data in Supplementary Table 1 are in Supplementary Table 2.

## Supplementary information

Supplementary Tables and Figures

## Data Availability

Supplementary Table 1 provides individual animal post-challenge data. All other data that support the findings of this study are available from the corresponding authors upon reasonable request.

## Supplementary information

**Supplementary Information** accompanies the paper on the *npj Vaccines* website (10.1038/s41541-019-0113-9).
