# Supplementary material for: Boosting BCG with proteins or rAd5 does not enhance protection against tuberculosis in rhesus macaques
Source: NPJ Vaccines. 2019 May 28;4:21. doi: 10.1038/s41541-019-0113-9 (PMC6538611; doi:10.1038/s41541-019-0113-9)
Supplement: Supplementary file 1 — Supplementary Tables and Figures [file 41541_2019_113_MOESM1_ESM.pdf]

Supplementary Table 1. Summary of macaque data post-challenge

	Monkey #	Infection date	Dose (CFU)	Pathology Score	Total CFU	Total FDG Activity	Days Post-Infection
Unvaccinated CFU, PET-CT, and Gross Pathology data from the unvaccinated animals previously published in reference 34	915	4/7/15	8	52	1,476,587	35047.66	76
	1215	4/7/15	8	53	63,521	14858.36	154
	1415	4/16/15	15	24	153,046	31589.70	98
	2315	4/16/15	15	36	18,620	1785.01	151
	6914	5/27/14	8	77	199,232		181
	7114	5/27/14	8	40	16,654	4605.39	195
	7313	7/30/13	16	93	2,012,810	58457.46	90
	7613	7/30/13	16	43	16,650	1185.02	162
	7614	5/27/14	8	97	675,163	256852.34	99
	8214	5/29/14	10	40	1,028,623	5590.44	186
	8514	5/29/14	10	66	3,106,670	64407.62	54
	8713	8/1/13	14	95	2,607,335	612414.93	110
BCG	7214	5/27/14	8	27	12,800	0.00	132
	7314	5/27/14	8	38	28,247	251.07	183
	7914	5/29/14	10	53	745,755	904.86	188
	8013	7/30/13	16	39	45,480	20490.53	128
	8114	5/29/14	10	22	6,131	335.34	193
	8213	7/30/13	16	40	98,620	155.64	186
	8813	8/1/13	14	43	34,410	6232.84	174
	9113	8/1/13	14	108	1,541,330	214269.35	90
BCG+M72: AS01E	7513	7/30/13	16	43	2,000,587	292653.67	99
	7514	5/27/14	8	18	1,550	0.00	183
	7714	5/27/14	8	50	634,437	248578.85	181
	7813	7/30/13	16	45	136,335	16684.00	160
	7814	5/29/14	10	45	8,961,627	93678.32	118
	8313	8/1/13	14	70	457,958	6680.50	155
	8414	5/29/14	10	19	16,158	3.35	186
	9013	8/1/13	14	17	8,020	104.22	169
BCG+H56: CAF01	1015	4/7/15	8	40	38,828	1406.80	120
	1515	4/7/15	8	36	374,466	15506.19	90
	1715	4/7/15	8	24	22,539	3818.20	176
	1915	4/16/15	15	31	954,112	16874.71	60
	3015	4/16/15	15	18	2,020	0.00	160
	3215	4/16/15	15	60	736,202	1882.80	104

Supplementary Table 1. Summary of macaque data post-challenge

	Monkey #	Infection date	Dose (CFU)	Pathology Score	Total CFU	Total FDG Activity	Days Post-Infection
BCG+Ad5 (empty)	1815	4/7/15	8	68	697,155	180860.64	97
	2015	4/7/15	8	32	97,226	3299.34	93
	2115	4/7/15	8	35	127,825	21942.85	78
	2215	4/16/15	15	56	3,050,252	53345.92	60
	2615	4/16/15	15	70	1,315,366	88499.83	137
	2815	4/16/15	15	98	1,801,548	126539.24	118
	2915	4/16/15	15	52	196,499	9112.62	109
BCG+Ad5 (M72)	6714	5/27/14	8	51	388,640	2415.80	115
	7014	5/27/14	8	104	16,527,398	2756004.63	134
	7213	7/30/13	16	37	47,110	8806.36	183
	7713	7/30/13	16	11	2,720	0.00	167
	8014	5/29/14	10	82	3,710,364	176559.62	116
	8513	8/1/13	14	24		8028.87	173
	8613	8/1/13	14	37	172,990	242.88	158
	8614	5/29/14	10	33	28,651	8115.68	195
BCG+Ad5 (EB)	6614	5/27/14	8	16	5,130	705.85	190
	6814	5/27/14	8	63	430,063	9720.19	146
	7413	7/30/13	16	31	6,540	3381.60	175
	7414	5/29/14	10	25	7,750	1159.99	195
	7913	7/30/13	16	51	1,688,828		120
	8314	5/29/14	10	72	3,791,001	226168.79	123
	8413	8/1/13	14	23	2,305	202.25	167
	8913	8/1/13	14	43	38,535	6110.32	165
BCG+Ad5 (4ag)	1115	4/7/15	8	31	740,705	7463.81	83
	1315	4/7/15	8	50	1,137,185	146884.20	135
	1615	4/7/15	8	29	206,099	2015.64	84
	2415	4/7/15	8	22	27,256	797.95	169
	2515	4/16/15	15	38	59,751	4189.44	95
	2715	4/16/15	15	36	48,985	2039.94	158
	3115	4/16/15	15	28	22,748	178.72	167

Supplementary Table 1. Summary of macaque data post-challenge. All macaques in this study, with identifying numbers, Mtb infection date and dose, gross pathology score, total thoracic bacterial burden, total lung FDG activity, and days to necropsy.

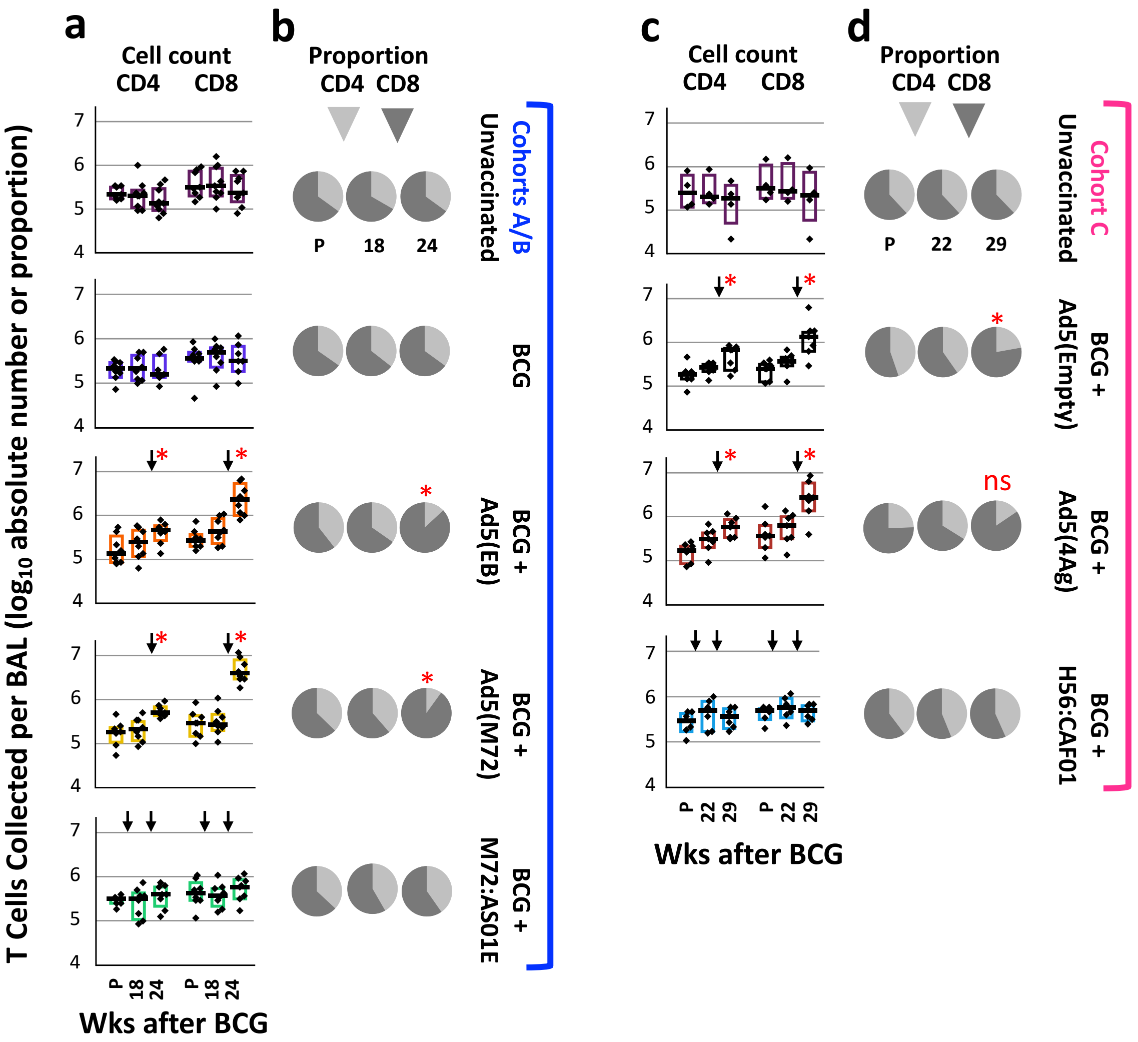

Supplementary Figure 1. Cellular kinetics in BAL after vaccination. The absolute number (cell count, **a, c**) and proportion (pies, **b, d**) of CD4 and CD8 T cells recovered from the BAL prior to vaccination (P), prior to boosting (18 or 22 weeks after BCG), or 4 weeks after the Ad5 or second protein boost (24 or 29 weeks after BCG) are shown for each experimental group (**a, b**, Cohorts A/B; **c, d,** Cohort C). Cell counts and are shown on a log_{10} scale. Black arrows indicate the time(s) of boosting. Dots represent individual animals, bars show the interquartile range with the median response indicated. *p≤0.05 compared to pre-vaccination (P) within the same group; # p≤0.05 compared to the most recent time point prior to boosting within the same vaccine group using two-tailed Student *t* test or permutation test (for pies).

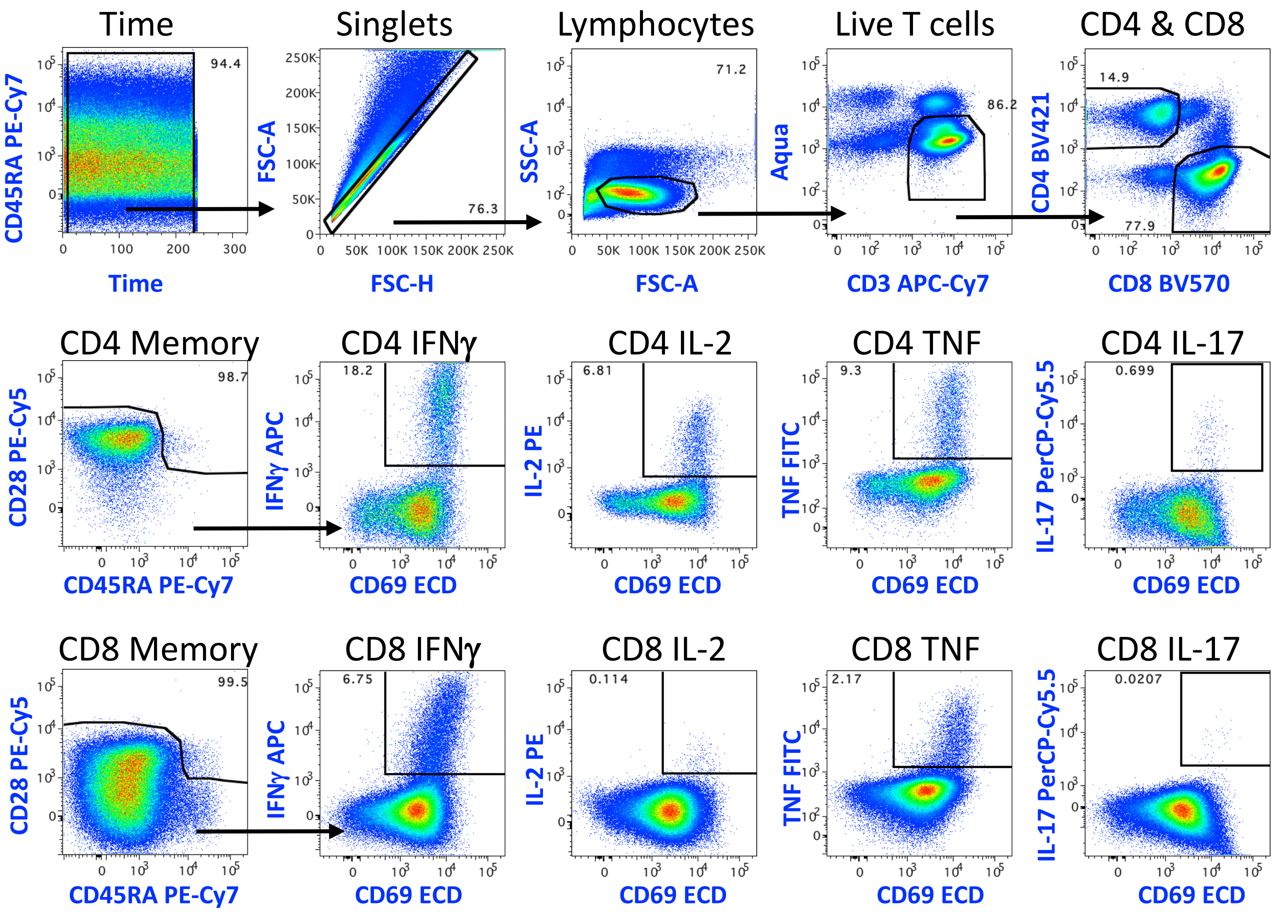

Flow Cytometry Panel for Modified BD LSR II					
**Ab specificity**	**Fluorochrome**	**Manufacturer**	**Catalog #**	**Clone**	**Lot**
Dead cells	Aqua Dead Cell Stain	ThermoFisher	L34957	--	not available
CD4	BV421	BioLegend	317433	OKT4	B156244
CD8	BV570	BioLegend	301037	RPA-T8	B157096
CD45RA	PE-Cy7	BD Biosciences	337167	L48	3016835
CD28	PE-Cy5	BD Biosciences	555730	CD28.2	2167822
CD69	ECD	Beckman Coulter	6607110	TP1.55.3	7620019
CD3	APC-Cy7	BD Biosciences	557757	SP34-2	28943
IFNg	APC	BD Biosciences	554702	B27	30523
IL-2	PE	BD Biosciences	554566	MQ1-17H12	41699
TNF	FITC	BD Biosciences	554512	Mab11	22119
CD103	AlexaFluor 680	Beckman Coulter	IM0318	2G5	not available
IL-17	PerCP-Cy5.5	BD Biosciences	560799	N49-653	3063916

Supplementary Figure 2. Gating strategy for BAL T cell cytokine production. Shown is a representative BAL sample from an animal immunized with BCG+Ad5(EB) stimulated with PPD 4 weeks after boosting. BAL cells were immunostained as described in materials and methods. After applying a time gate, singlet gate, and lymphocyte gate, live $CD3^+$ T cells were gated followed by selection for $CD4^+$ and $CD8^+$ T cells. Although BAL contains few naïve T cells, a memory gate (non-$CD28^+CD45RA^+$) was applied to $CD4^+$ and $CD8^+$ T cells. Cytokine-producing memory T cells were gated against CD69, as shown.

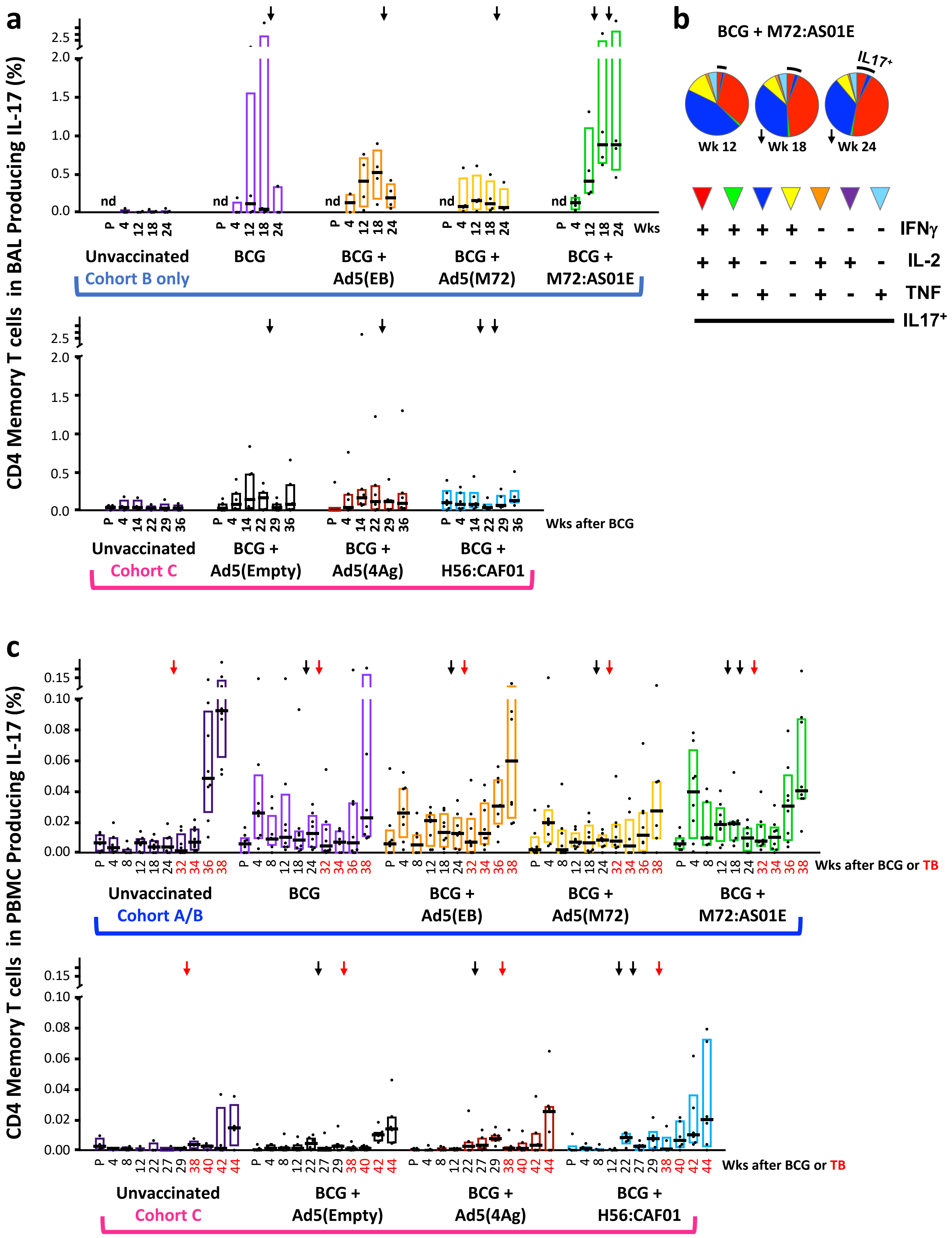

Supplementary Figure 3. IL-17 responses in BAL after vaccination and in PBMC after vaccination and challenge. **a**) The frequency of memory CD4 T cells in the BAL producing IL-17 in response to PPD at the indicated weeks after BCG immunization and subsequent boosting for Cohort B (top) and Cohort C (bottom). IL-17 was not measured for Cohort A or at the pre-vaccination time point for Cohort B; nd, not done. Black arrows indicate the time of boosting, dots represent individual animals, bars show the interquartile range with the median response indicated. **b**) Pie slices represent the fraction of the total CD4 memory T cell response to PPD comprising any combination of IFNg, IL-2, or TNF, with or without IL-17 production (black arc) for BCG + M72:AS01E immunized animals (Cohort B only) before or after each boost (arrows). **c**) The frequency of memory CD4 T cells in PBMC producing IL-17 in response to PPD the indicated weeks before and after immunization and boosting, and after Mtb challenge (red-numbered weeks) is shown for each experimental group (Cohorts A/B, top; Cohort C, bottom). IL-17 data are shown for PPD stimulation as peptide-induced IL-17 was low to undetectable before and after boosting (not shown). Black and red arrows indicate the time of boosting and challenge, respectively. Dots represent individual animals, bars show the interquartile range with the median response indicated.

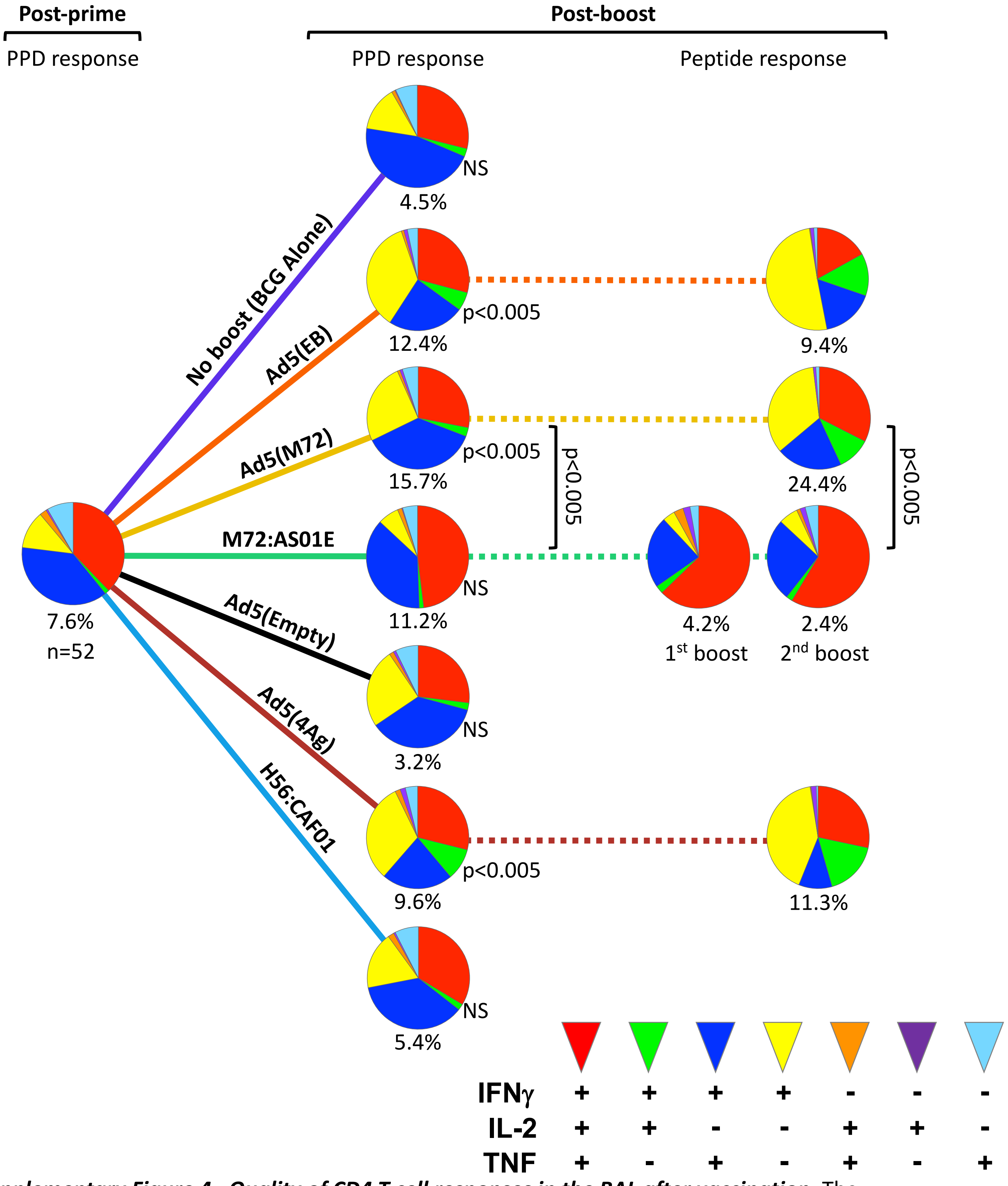

Supplementary Figure 4. Quality of CD4 T cell responses in the BAL after vaccination. The multifunctionality of the CD4 T cell cytokine response (Fig. 2) was analyzed at the peak after priming (12-14 weeks after BCG; Post-prime) for all animals receiving BCG (n=52) and 4 weeks after boosting (24 or 29 weeks after BCG; Post-boost) for animals in each individual vaccine group (n=6-8). Pie charts represent the fraction of the total cytokine response comprising any combination of IFNg, IL-2, or TNF produced in response to PPD, or peptides for vaccine groups with frequencies (shown beneath each pie) greater than those prior to boosting. P values indicate qualitative differences in the PPD response before and after boosting, or the difference in quality between two boosting regimens [Ad5(M72) and M72/AS01$_E$] in response to PPD or M72 peptides following boosting using a permutation test.

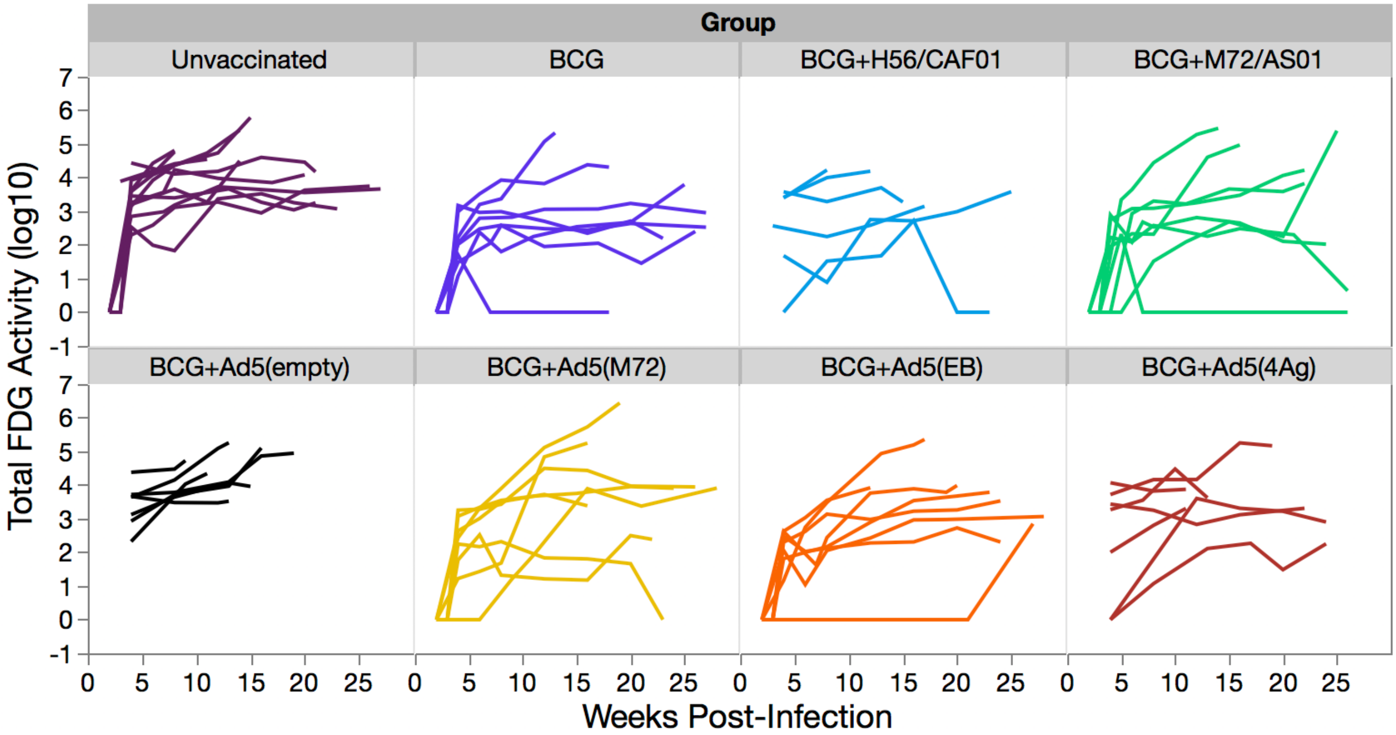

Supplementary Figure 5. Serial PET scans of each animal in each vaccine group. Total lung FDG activity is shown (log10 scale) for each animal during the course of infection. Most, but not all, animals had pre-infection scans.

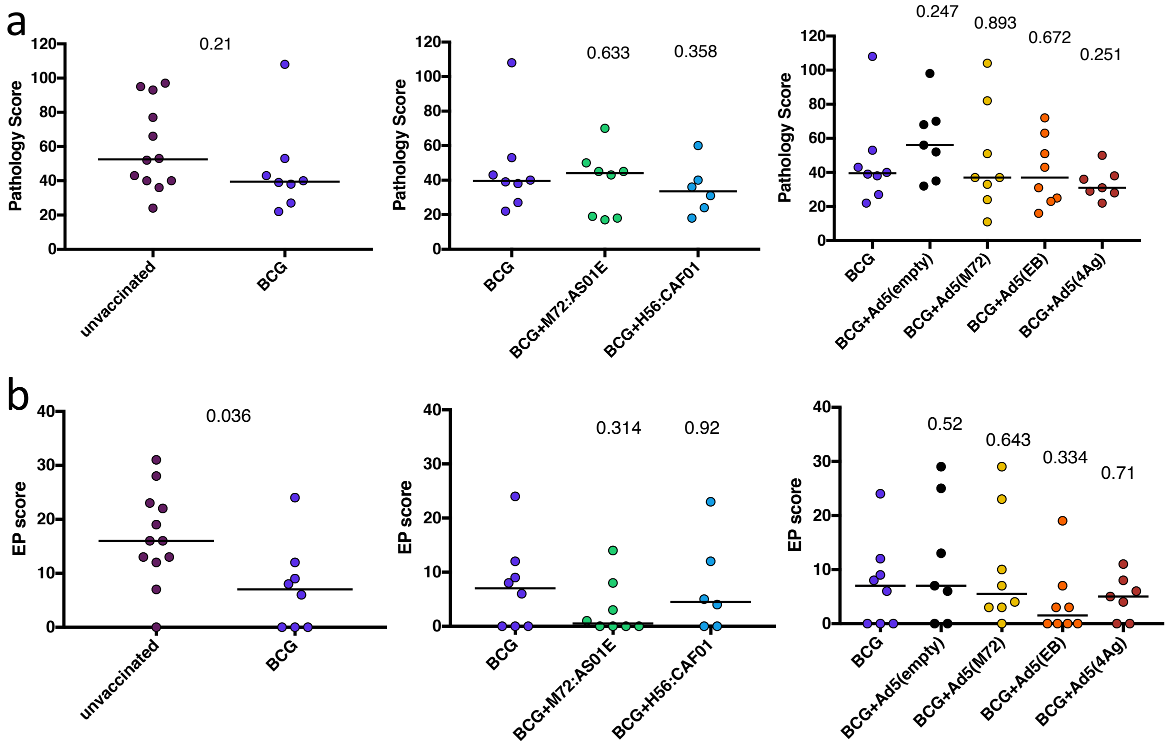

Supplementary Figure 6. Gross Pathology and extrapulmonary disease scores. (**a**) Gross pathology was quantified at necropsy using our published scoring system [34], which includes all lung, lymph node and extrapulmonary disease observed grossly during detailed necropsy. (**b**) Extrapulmonary score includes gross disease at necropsy in all extrapulmonary organs (e.g. spleen, liver, kidney, paracostal or paravertebral abscess) as well as whether samples from these organs grew Mtb, as described in [34]. For each graph, the comparator group was BCG alone. A Kruskal-Wallis test was performed and uncorrected Dunn's test p-values are reported. Dots represent individual animals and lines represent medians.

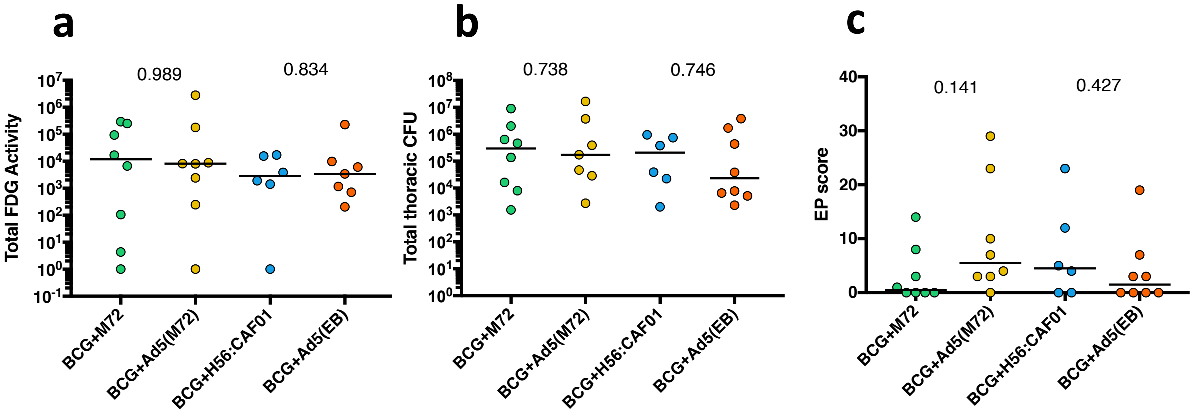

Supplementary Figure 7. Vaccination with antigens delivered as protein and adjuvant result in similar infection outcomes to those antigens delivered in the context of Ad5. Total FDG activity in lung (**a**), total thoracic (lung + lymph node) bacterial burden (**b**), extrapulmonary disease scores (**c**) were compared between BCG boosted by M72/AS02 and BCG boosted by Ad5(M72) and between BCG boosted by H56/CAF01 and BCG boosted by Ad5(EB). Uncorrected Dunn's test p-values are reported. Dots represent individual animals and lines represent medians.

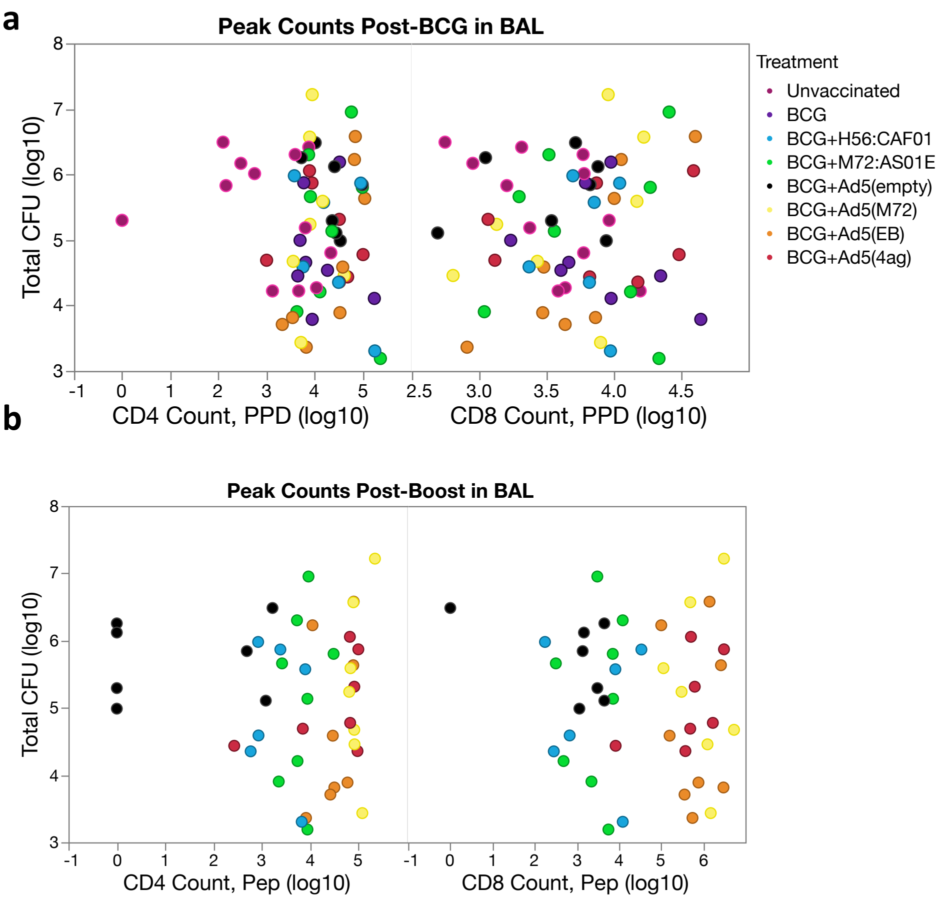

Supplementary Figure 8. BAL T cell responses and infection outcome. No correlation was found between the peak post-BCG vaccination CD4 or CD8 T cell response (numbers of cells responding) in BAL to PPD (A) or post-boost response to vaccine specific peptides (B) for each animal and total thoracic bacterial burden at necropsy. For B, only boosted animals were included in the analysis. Spearman's rho was calculated for each pair of variables shown in the graphs with no statistically significant correlations. Dots represent individual animals.

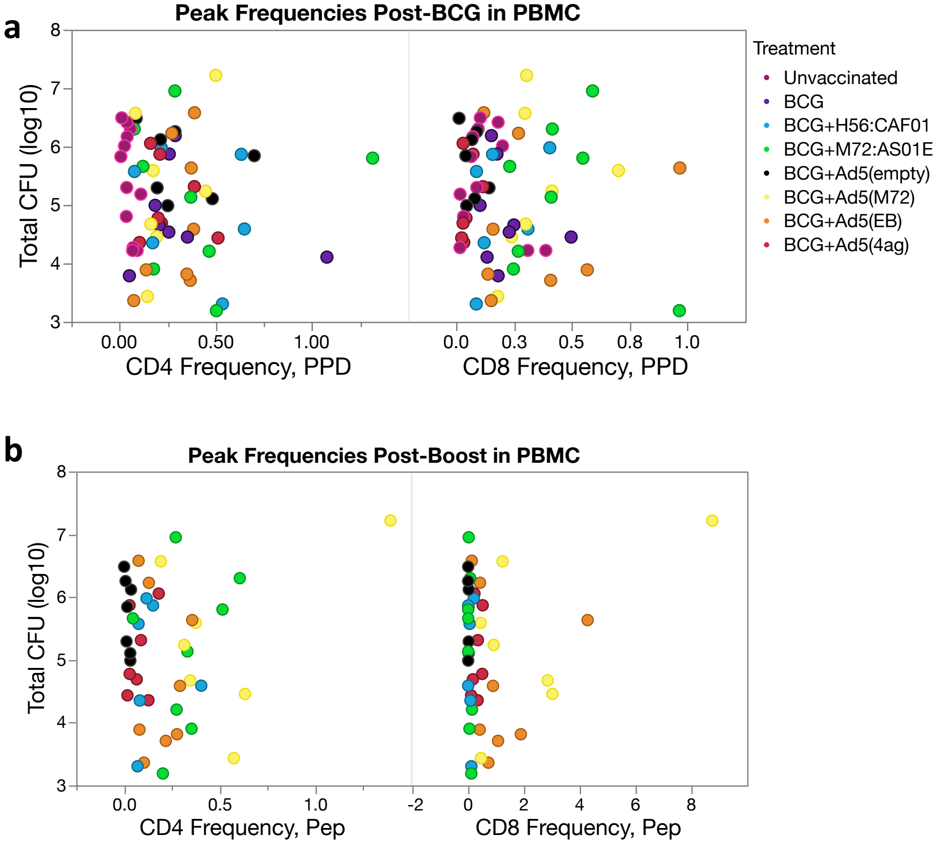

Supplementary Figure 9. PBMC T cell responses and infection outcome. No correlation between the peak frequency of CD4 or CD8 memory T cells in blood responding to PPD post-BCG vaccination (A) or post-boost to vaccine specific peptides (B) for each animal and total thoracic bacterial burden at necropsy. For B, only boosted animals were included in the analysis. Spearman's rho was calculated for each pair of variables shown in the graphs with no statistically significant correlations. Dots represent individual animals.

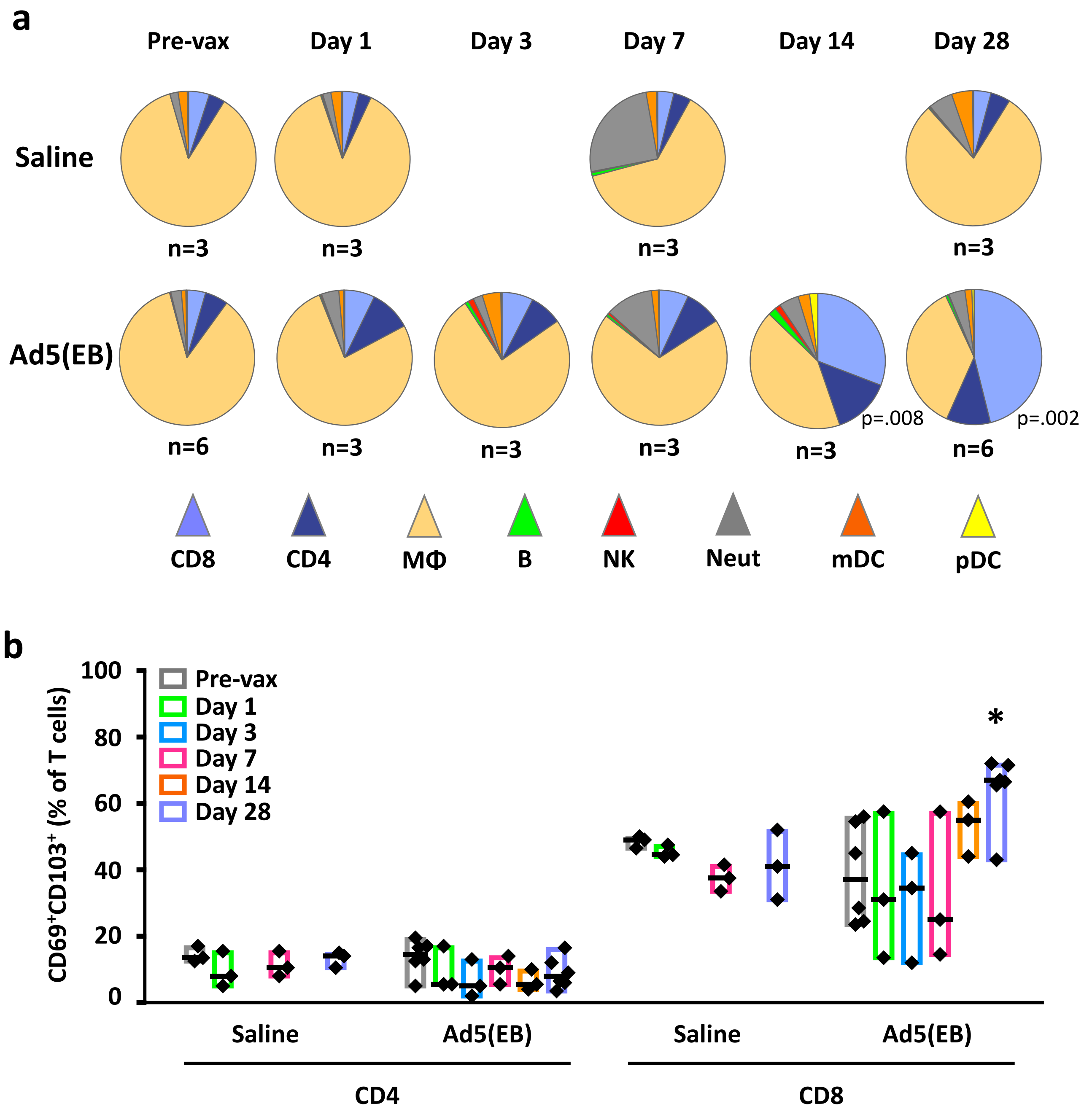

Supplementary Figure 10. Cellular kinetics in BAL following AE Ad5 immunization. The composition of leukocytes present in the BAL after AE administration of saline (n=3) or Ad5(EB) (n=6) were analyzed by flow cytometry at the following time points: pre-vaccination (pre-vax), day 1, day7, and day 28 (saline and half Ad5(EB) animals) or pre-vax, day 3, day 14, and day 28 (half Ad5(EB) animals). (**a**) Pies show the proportion of each indicated cell type before and after AE immunization; Ad5(EB) animals sampled on different schedules are shown separately. (MΦ: macrophages, B: B cells; NK: natural killer cells; Neut: neutrophils, mDC: myeloid dendritic cells; pDC: plasmacytoid dendritic cells). P values indicate differences in cellular composition from pre-vax using a permutation test. (**b**) Percent of total CD4 or CD8 T cells expressing CD69 and CD103 in the BAL before and after AE Ad5(EB) immunization. *$p \leq 0.05$ compared to pre-vax within the same group and same T cell subset using a two-tailed Student *t* test.

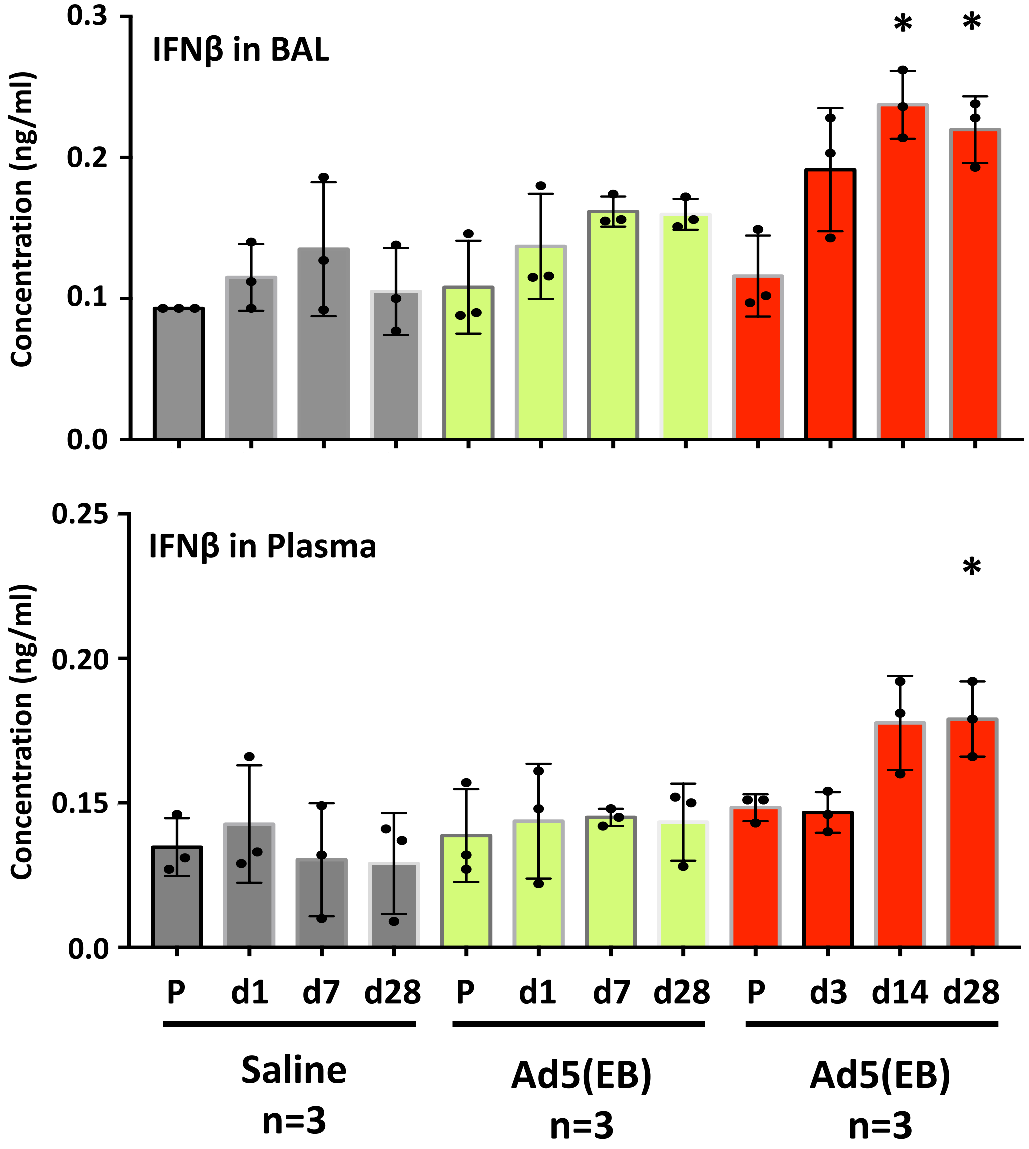

Supplementary Figure 11. Cytokines elicited by AE Ad5 administration. Cytokine detection in concentrated BAL wash fluid or plasma from animals after AE administration of saline (n=3) or Ad5(EB) (n=6 with half the animals sampled at pre-vaccination (P), d1, d7, and d28 and the other half sampled at P, d3, d14, and d28). Shown are the amount of Interferon beta (IFN-b) in the BAL fluid (top) and plasma (bottom) at the indicated time points after immunization. *$p \leq 0.05$ compared to pre-vax within the same group using a two-tailed Student *t* test. I removed the IL-Ra panel as requested by reviewer

Supplementary Table 2. Descriptive Statistics of Outcome Variables

Necropsy Scores

	Total			Lung			Lymph Nodes		
	n	Median	IQR	n	Median	IQR	n	Median	IQR
**unvaccinated**	12	52.5	49	12	18.5	31.25	12	23	6.5
**BCG**	8	39.5	20.75	8	10	18.5	8	22	7
**BCG+M72:AS01**	8	44	30.5	8	23.5	26.5	8	16	5.25
**BCG+H56:CAF01**	6	33.5	22.5	6	9	8	6	18.5	6.25
**BCG+Ad5(empty)**	7	56	35	7	24	25	7	24	3
**BCG+Ad5(M72)**	8	37	48	8	13	30.5	8	21	9.75
**BCG+Ad5(EB)**	8	37	36.5	8	10.5	21.5	8	21	8.25
**BCG+Ad5(4ag)**	7	31	10	7	9	5	7	18	6

Total CFU

	Total Thoracic			Lung			Lymph Nodes		
	n	Median	IQR	n	Median	IQR	n	Median	IQR
**unvaccinated**	12	437197.5	1848909	12	233664.4	810387	12	98448	867645
**BCG**	8	39945	567310	8	2135	29795	8	26409	574239
**BCG+M72:AS01**	8	297146.5	1648995	8	97316.25	395485	8	104128	442519
**BCG+H56:CAF01**	6	206647	773270	6	18750.5	112123	6	188014	612271
**BCG+Ad5(empty)**	7	697155	1673723	7	299041	1234381	7	164986	380900
**BCG+Ad5(M72)**	7	172990	3681713	7	16860	987188	7	172345	2673295
**BCG+Ad5(EB)**	8	23142.5	1368654	8	3232.5	574927	8	20945	511715
**BCG+Ad5(4ag)**	7	59751	713449	7	26025	94411	7	45505	644367

Extrapulmonary and Total Lung FDG Activity

	Extrapulmonary Score			Lung FDG Activity (12 weeks)			Lung FDG Activity (Pre-Necropsy)		
	n	Median	IQR	n	Median	IQR	n	Median	IQR
**unvaccinated**	12	16	10.5	10	7450.238	37544.6	11	31590	59802.2
**BCG**	8	7	11.25	8	403.3886	5152.95	8	620.1	16746.6
**BCG+M72:AS01**	8	0.5	6.75	8	1153.678	30642.1	8	11682	209825
**BCG+H56:CAF01**	6	4.5	14.75	5	567.7377	10050.2	6	2850.5	14793.2
**BCG+Ad5(empty)**	7	7	25	6	12011.84	39413.6	7	53346	117427
**BCG+Ad5(M72)**	8	5.5	16.75	8	4972.046	59257.3	8	8072.3	133835
**BCG+Ad5(EB)**	8	1.5	6	8	864.866	7466.34	7	3381.6	9014.34
**BCG+Ad5(4ag)**	7	5	8	7	4003.121	6789.65	7	2039.9	6665.86

Supplementary Table 2. Descriptive statistics of outcome variables. Median, interquartile range, and sample size of necropsy score, total CFU, extrapulmonary score, and total lung FDG activity by treatment group.
